# Targeting the HECTD3-p62 axis increases the radiosensitivity of triple negative breast cancer cells

**DOI:** 10.1038/s41420-024-02154-5

**Published:** 2024-11-01

**Authors:** Maobo Huang, Wenjing Liu, Zhuo Cheng, Fubing Li, Yanjie Kong, Chuanyu Yang, Yu Tang, Dewei Jiang, Wenhui Li, Yudie Hu, Jinhui Hu, PemaTenzin Puno, Ceshi Chen

**Affiliations:** 1 Kunming Institute of Zoology, Chinese Academy of Sciences, Kunming, 650201 China; 2 The First People’s Hospital of Kunming City (The Affiliated Calmette Hospital of Kunming Medical University), Kunming, 650224 China; 3 The Third Affiliated Hospital, Kunming Medical University, Kunming, 650118 China; 4 Kunming College of Life Sciences, University of the Chinese Academy of Sciences, Kunming, 650204 China; 5 Academy of Biomedical Engineering, Kunming Medical University, Kunming, 650500 China; 6 Biobank, Shenzhen Second People’s Hospital/ the First Affiliated Hospital of Shenzhen University Health Science Center, Shenzhen, 518035 China; 7 The First Hospital of Hunan University of Chinese Medicine, Changsha, 410007 Hunan China; 8 State Key Laboratory of Phytochemistry and Plant Resources in West China, Kunming Institute of Botany, Chinese Academy of Sciences, Kunming, 650201 Yunnan China

**Keywords:** Breast cancer, Macroautophagy, Radiotherapy

## Abstract

Triple negative breast cancer is the most malignant subtype of breast cancer and current treatment options are limited. Radiotherapy is one of the primary therapeutic options for patients with TNBC. In this study, we discovered that the E3 ubiquitin ligase, HECTD3, promoted TNBC cell survival after irradiation. HECTD3 collaborated with UbcH5b to promote p62 ubiquitination and autophagy while HECTD3 deletion led to p62 accumulation in the nucleus in response to irradiation, thus inhibiting RNF168 mediated DNA damage repair. Furthermore, the HECTD3/UbcH5b inhibitor, PC3-15, increased the radiosensitivity of TNBC cells by inhibiting DNA damage repair. Taken together, we conclude that HECTD3 promotes autophagy and DNA damage repair in response to irradiation in a p62-denpendent manner, and that inhibition of the HECTD3-p62 axis could be a potential therapeutic strategy for patients with TNBC in addition to radiotherapy.

## Introduction

Triple negative breast cancer (TNBC), accounting for 15–20% of breast cancer cases, is the most malignant subtype of breast cancer with a poor prognosis [1]. Radiotherapy (RT) is the primary therapeutic strategy for patients with TNBC. However, radio-resistance of cancer cells remains a major limitation. Moreover, high-energy radiation inevitably causes side effects such as lung fibrosis and heart failure [2–4]. Therefore, it is important to improve the effects of low-dose RT.

Irradiation (IR) eradicates tumor cells mainly by inducing lethal DNA double-strand breaks (DSBs), whereas DNA damage repair (DDR) contributes to resistance [5]. Non-homologous end joining (NHEJ) and homologous recombination (HR) are two major mechanisms responsible for the timely and efficient repair of DSBs [6]. DSBs induce H2AX phosphorylation at S139, known as γH2AX, which provides a docking platform for mediating DNA damage and checkpoint 1 (MDC1) binding [7]. Subsequently, RNF8 is recruited to DSBs via interaction with MDC1 [8]. RNF8 then recruits another E3 ligase, RNF168, to catalyze the formation of K63-linked polyubiquitin chains on the K13-15 of H2A and H2AX [9]. This ubiquitination signaling is necessary for the further recruitment of downstream effectors of the DDR pathway, such as 53BP1 and the BRCA1 complex, and hence determines the cellular response to DNA damage through NHEJ and HR, respectively [10, 11]. RNF168 is essential for DDR, depending on its E3 activity [12].

It has been reported that autophagy is activated by IR [13]. Autophagy is widely known to promote cancer cell survival from IR and induce radio-resistance [14–16]. Autophagy-mediated HP1 degradation is required for HR [17] and autophagy deficiency impairs DSB repair [18]. More importantly, autophagy directly decreases the levels of p62, an autophagy cargo that plays a key role in DDR. The nuclear p62 promotes the degradation of critical HR proteins, FLNA and RAD51, and inhibits RNF168 mediated histone ubiquitination, thus inhibiting DDR [19, 20]. Additionally, p62 accumulation in aged cells and tissues, characterized by impaired autophagy, dramatically reduces the efficiency of DDR [21]. These reports indicate an important role for autophagy in DDR.

HECTD3, a HECT family E3 ligase belonging to the HECT family, plays a vital role in drug resistance and metastasis by ubiquitinating several substrate proteins. HECTD3 is highly expressed in multiple cancers, including breast cancer, gastric cancer, ovarian cancer, and glioma [22]. HECTD3 facilitates breast cell survival from cisplatin and TRAIL by ubiquitinating MALT1 with non–K48-linked polyubiquitin chains and Caspase-8 with K63-linked polyubiquitin chains [23, 24]. HECTD3 promotes esophageal squamous cell carcinoma (ESCC) growth and cell survival by suppressing Caspase-9 activation [25]. HECTD3 inhibits apoptosis in gastric cancer cells by polyubiquitinating c-Myc with K29 linked polyubiquitin chains [26]. Depletion of HECTD3 sensitizes glioma cells to radiation by regulating LKB1 ubiquitination and downregulating ZEB1 expression [27]. Recently, we demonstrated that HECTD3 promotes cancer metastasis by ubiquitinating IKKα and increasing kinase activity in endothelial cells [28]. Additionally, HECTD3 can block NLRP3 inflammasome activation independent of its E3 ligase activity [29]. These results imply that HECTD3 is a potential therapeutic target for cancer therapy.

In this study, we showed that HECTD3 promoted autophagy and DDR by ubiquitinating p62 in TNBC cells. HECTD3 promotes TNBC cell survival from IR by activating autophagy and facilitating DSB repair in a p62 dependent manner. More importantly, we demonstrated that the small-molecule HECTD3/UbcH5b inhibitor, PC3-15, inhibited IR-induced autophagy and blocked DDR in TNBC cells. PC3-15 increases the sensitivity of TNBC cells to RT in vitro and in vivo. This study reveals a new mechanism of p62 ubiquitination via HECTD3 and provides a potential strategy for the development of PC3-15 as a radiosensitizer.

## Results

### The UbcH5b/HECTD3 inhibitor PC3-15 increases the radiosensitivity of TNBC cells

**Fig. 1:**
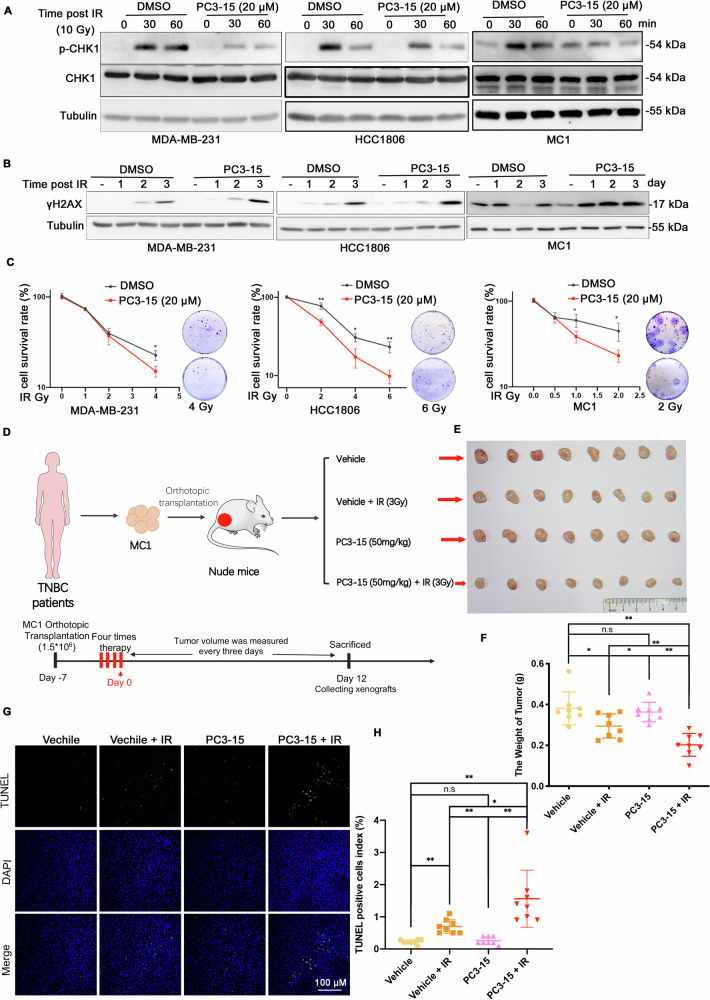
PC3-15 increases the sensitivity of TNBC cells. **A** PC3-15 inhibits pCHK1 levels in TNBC cells. MDA-MB-231, HCC1806 and MC1 cell lines were pretreated with PC3-15 (20 μm) for 2 h before IR (10 Gy) and harvested for WB at 0, 30, and 60 min. **B** PC3-15 pretreatment increased the accumulation of ${\rm{\gamma }}$H2AX after IR in both MC1 and HCC1806 cells. MC1 and HCC1806 cells were pretreated with PC3-15 (20 μm) for 2 h before IR (10 Gy) and harvested for WB at 1-3 days. **C** PC3-15 pretreatment decreased survived clones with the increase of IR. Clonogenic survival in response to IR of MC1 and HCC1806 cells pretreated with PC3-15 (20 μm) for 2 h. The column represents the number of clones when only PC3-15 (20 μm) is pretreated. Data are mean ± SD. Statistical analysis was performed using two-tailed unpaired t-tests. Data are representative of three independent experiments. *p < 0.05 and **p < 0.01; n.s, not significant. **D**. Schematic diagram of characterization of PC3-15 combined with irradiation therapy in TNBC patient-derived xenografts. **E**–**H** PC3-15 increased radiotherapy sensitivity of MC-1 in vivo. MC1 cells were injected into the fat pat of nude mice. Mice carried MC1 xenografts were randomly distributed into four groups equally when the tumor size reached around 70 mm^3^ and were administrated with 50 mg/kg PC3-15 or vehicle following with and without IR (3 Gy×4) by intraperitoneal injection. After 12 days of 4 times therapy, the mice were euthanized, the image of tumors was shown (**E**), and the tumor weight (**F**) and TUNEL positive ratio (**G**, **H**) were recorded. Data are mean ± SD. Statistical analysis was performed using two-tailed unpaired t-tests. *P < 0.05 and **P < 0.01; ns, not significant. Scale bar, 100 μm.

In our previous study, we reported that PC3-15 is a HECTD3/UbcH5b inhibitor, although the exact mechanism was unclear, and we found PC3-15 increased lapatinib sensitivity of TNBC cells by inhibiting autophagy [30]. Here, we treated HCC1806 cells with IR and PC3-15 in combination or alone. Interestingly, we observed that PC3-15 also inhibited IR-induced autophagy, as evidenced by PC3-15 cells had reduced LC3II protein levels in response to IR and inhibited autophagy-related signaling pathway by RNA-seq analysis (Fig. S1A, B). It was recently reported that autophagy deficiency impairs HR but not NHEJ by reducing CHK1 phosphorylation [18]. As we expected, we found that PC3-15 effectively reduced IR-induced activation of CHK1 in several TNBC cell lines (Fig. 1A). These results imply that PC3-15 inhibits IR-induced DDR.

Then, we test whether PC3-15 could enhance IR therapy sensitivity of TNBC. First, we confirmed that PC3-15 pretreatment sensitized TNBC cells to IR treatment, as evidenced by the increased accumulation of ${\rm{\gamma }}$H2AX after IR (Fig. 1B) and decreased survival clones (Fig. 1C).

Next, we established a TNBC patient-derived xenograft (PDX) model by injecting MC1 cells into the fat pads of 6 weeks female nude mice to evaluate the therapeutic efficacy of PC3-15 in combination with IR in vivo. We selected a low dose for each treatment (3 Gy/treatment, 12 Gy in total) (Fig. 1D). Remarkably, the combination of PC3-15 and IR significantly inhibited tumor growth with the highest efficiency, and the mouse body weight did not show any difference (Fig. 1E, F and S1C). Consistently, we detected the highest proportion of TUNEL-positive apoptotic cells in PC3-15-IR joint-treated tumors (Fig. 1G, H). These results indicated that pre-administration of PC3-15 could improve the therapeutic benefits of IR therapy against TNBC.

### HECTD3 promotes cancer cell survival from IR

**Fig. 2:**
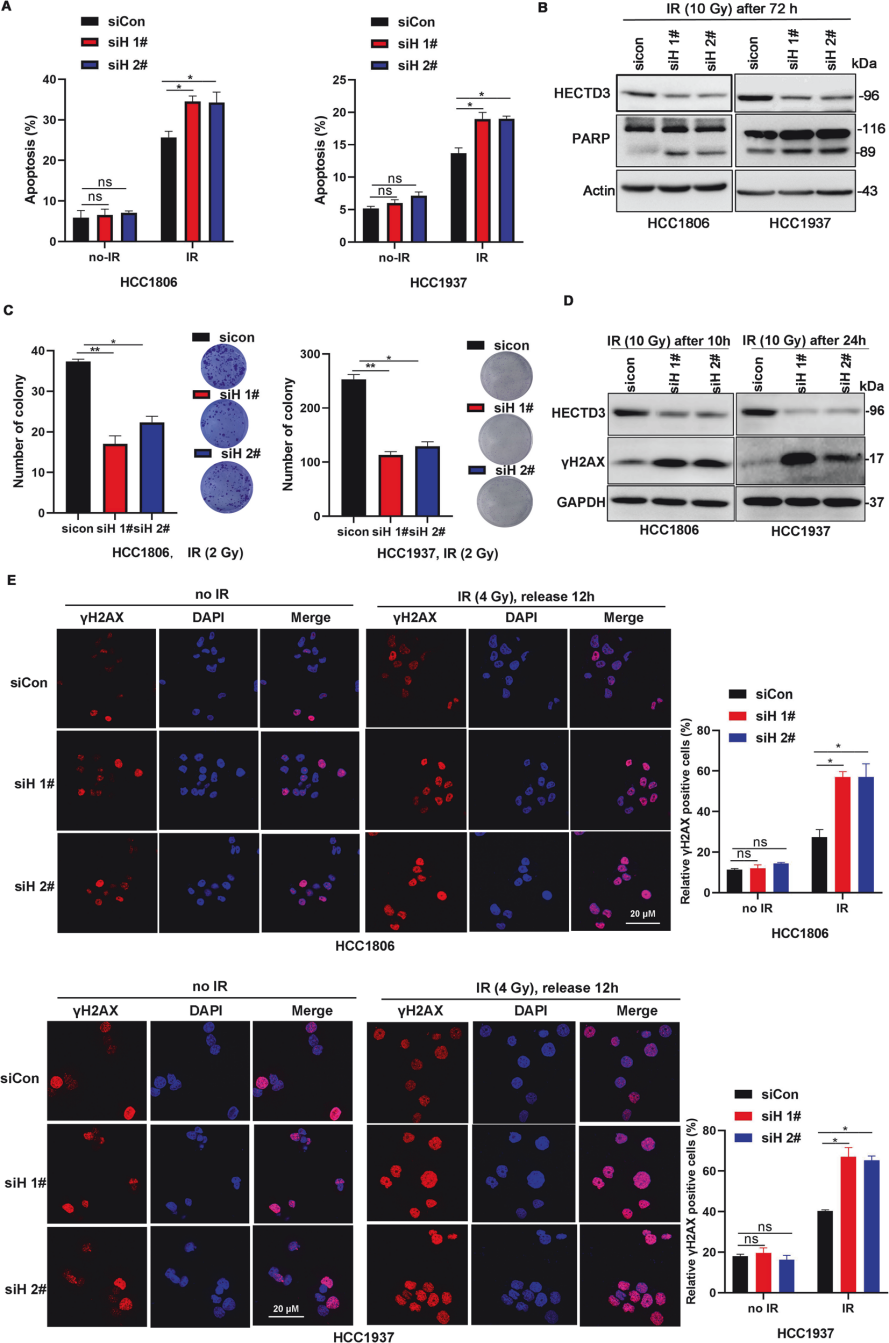
HECTD3 KD sensitizes TNBC cells to IR. **A** HECTD3 knockdown increased IR-induced apoptosis in HCC1806 and HCC1937 cell lines. Cells were transfected with siCon or siHECTD3, treated with IR (10 Gy), and harvested for annexin V/PI staining analysis by flow cytometry at 72 h point post-IR. *p < 0.05 and ns, not significant. **B** HECTD3 knockdown increased the cleavage of PARP after IR in both HCC1806 and HCC1937 cell lines. HCC1806 and HCC1937 cell lines transfected with siCon or siHECTD3 were treated with IR (10 Gy) and harvested for Western blotting analysis at 72 h point post-IR. **C** HECTD3 knockdown inhibited colony formation after IR. HCC1806 and HCC1937 cell lines transfected with siCon or siHECTD3 were irradiated (2 Gy). Colonies containing >40 cells were counted after 10–14 days. *p < 0.05 and **p < 0.01; ns, not significant. **D** HECTD3 knockdown increased the accumulation of ${\rm{\gamma }}$H2AX after IR in both HCC1806 and HCC1937 cell lines. HCC1806 and HCC1937 cells transfected with siCon or siHECTD3 were treated with IR (10 Gy) and harvested for Western blotting analysis at 10 or 24 h post-IR. **E** HECTD3 knockdown displays more IR-induced γH2AX foci after IR treatment in HCC1806 and HCC1927 cells. Cells transfected with siCon or siHECTD3 were treated with IR (10 Gy), after 12 h or 24 h, the cells were immunostained with anti-γH2AX (red) and DAPI (blue) and analyzed using a confocal microscopy. The cells containing >5 γH2AX foci were recorded as γH2AX positive cells, whose percentage was averaged from at least 100 cells. The data are displayed as mean plus SD of three counts, and statistical significance was calculated and represented as the *P*-value. *p < 0.05 and ns, not significant. Scale bars, 20 μm.

The above evidence prompted us to analyze the role of HECTD3 in cancer cells showing resistance to IR. To this end, we knocked down endogenous HECTD3 in HCC1806 and HCC1937 human TNBC cells using two different siRNAs and treated them with IR. Compared with control cells, HECTD3 knockdown (KD) cells were more prone to IR-induced cell death, as determined by apoptosis and PARP cleavage assays (Fig. 2A, B). Consistent with this finding, HECTD3 KD significantly reduced the number of colonies after IR (Fig. 2C). Notably, HECTD3 KD led to the accumulation of γH2AX, a DNA damage marker, in both cell lines (Fig. 2D, E). Additionally, we generated a *Hectd3* whole-body KO mouse model [28, 31]. We treated wild-type (WT) and *Hectd3* whole-body KO mice with IR (7 Gy, a lethal dose that kills all WT mice within approximately 3 weeks) and found that the survival time of the *Hectd3* KO group was significantly shorter than that of the WT mice (Figures S2A, B). These results indicate that HECTD3 could promote IR-induced DDR and cancer cell and mice survival from IR.

### HECTD3 facilitates DNA damage repair by activating ATR-CHK1 signaling

**Fig. 3:**
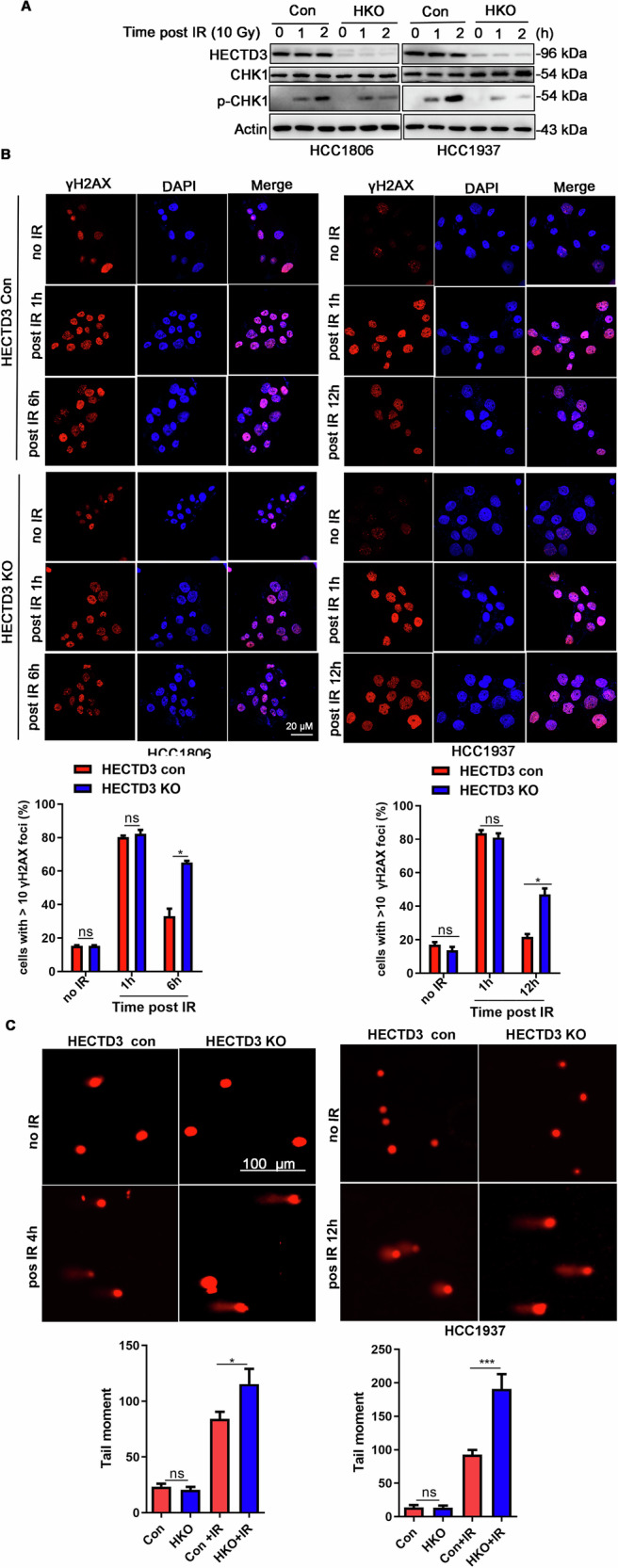
HECTD3 facilitates IR-induced DNA damage repair. **A** HECTD3 KO reduced pCHK1 (S345) protenin levels. HECTD3 (Con), HECTD3 (KO) and p62 knockdown/HECTD3 KO HCC1806 and HCC1937 cells were irradiated with 10 Gy. CHK1 and pCHK1 were detected by WB. **B** HECTD3 KO HCC1806 and HCC1937 cells show a decrease of IR-induced γH2AX foci compared to control cells. At 0, 1, 6, 12 h after irradiation (10 Gy), the cells were immunostained with anti-γH2AX (red) and DAPI (blue) and analyzed using a confocal microscopy. The cells containing >10 γH2AX foci were recorded as γH2AX positive cells. Average percentage values from at least 100 cells were recorded. The data are displayed as the mean plus SD of three counts, and statistical significance was calculated and represented as the *P*-value. *P < 0.05; ns, not significant. Scale bars, 20 µm. **C** HECTD3 enhances DNA damage repair in HCC1806 and HCC1937 cells, as measured by the COMET assay. Cells were treated with IR (10 Gy) for 4 h or 12 h and COMET images were captured using a fluorescence microscopy. The tail moment was calculated as the tail length multiplied by the tail DNA percentage in at least 100 cells. The data are displayed as the mean plus SD of three counts, and statistical significance was calculated and represented as the *P*-value. *P < 0.05; ***P < 0.001 and ns, not significant. Scale bars, 100 µm.

Next, we sought to test whether HECTD3 regulates DSB repair. To this end, we firstly generated HECTD3 KO breast cancer cell lines using the CRISPR-Cas9 system, as described previously [32]. Due to the defects in DNA damage repair in most TNBC, there is greater reliance on ATR-CHK1 pathway signaling following DNA damage, including IR‐induced DSBs [33]. Thus, we examined CHK1 phosphorylation after IR treatment. As shown in Fig. 3A, pCHK1 levels were significantly reduced in HECTD3 KO cells. Next, we measured the γH2AX foci at different time points (1, 6, and 12 h) after IR in HCC1806 and HCC1937 cells using immunofluorescence staining. HECTD3 KO resulted in significantly increased numbers of γH2AX+ cells compared with controls at 6 h in HCC1806 cells and at 12 h in HCC1937 cells, whereas the numbers of γH2AX foci at 1 h showed no difference in either cell line (Fig. 3B), indicating that HECTD3 KO decreased the DSB repair ability in TNBC cells.

To further verify that HECTD3 decreased DNA damage, a COMET assay was performed at 4 h post-IR. The tail moment was calculated as tail length × tail DNA% to evaluate the number of DNA breaks. IR-induced DNA breaks in TNBC cells were significantly higher in HECTD3 KO cells than in control cells (Fig. 3C). Taken together, these data confirm that HECTD3 deficiency impairs IR-induced DSB repair by inhibiting ATR-CHK1 sihnaling pathway.

### HECTD3 interactes with and ubiquitylates p62

**Fig. 4:**
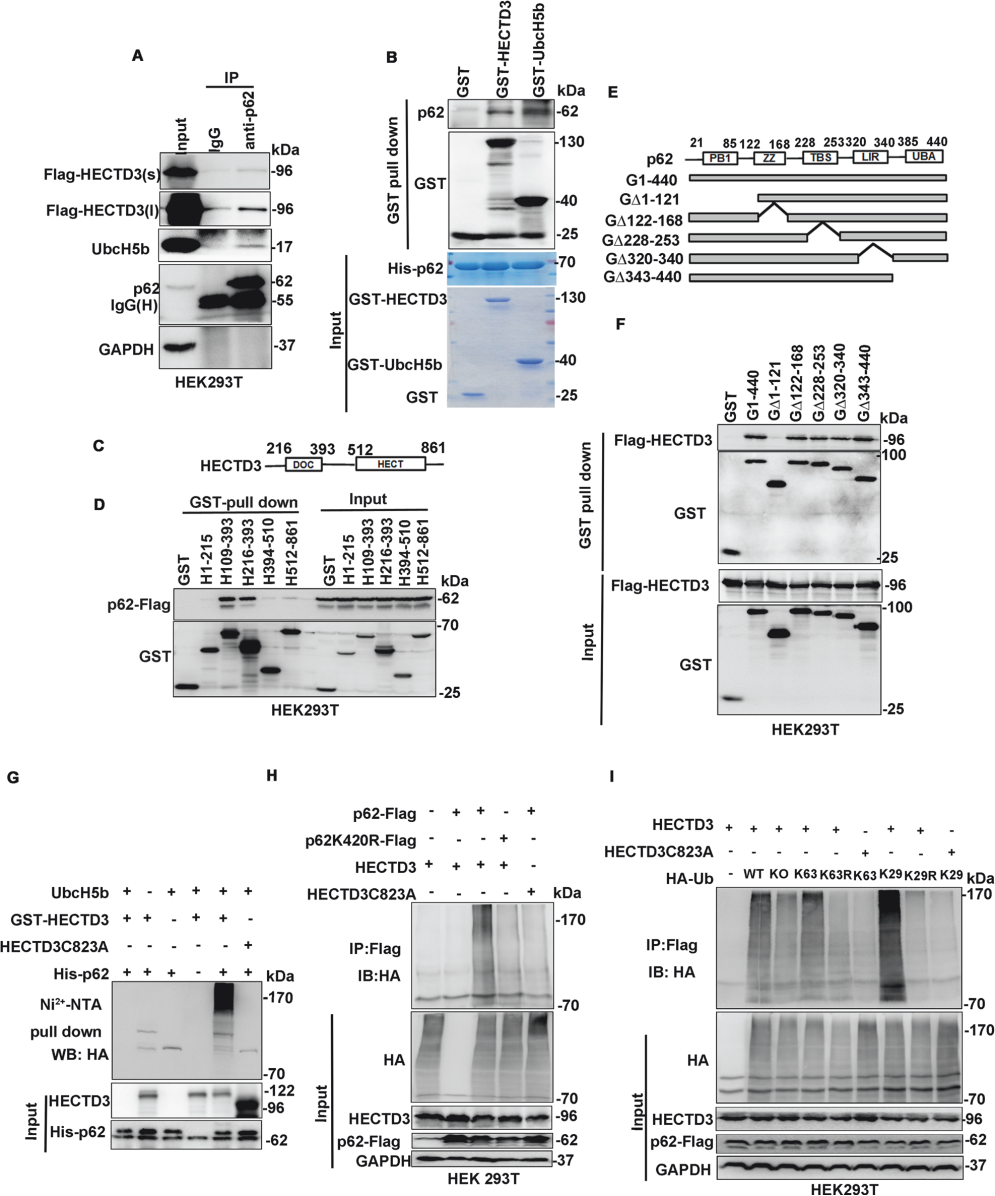
HECTD3 interacts with and ubiquitylates p62. **A** Co-immunoprecipitation of endogenous p62, UbcH5b and exogenously expressed Flag-HECTD3 in HEK293T cells. IgG was used as the negative control for anti-p62 Ab. S and L mean short and long exposure. **B** HECTD3 and UbcH5b directly interact with p62, as measured by GST pulldown assays. Purified GST fused HECTD3 and UbcH5b proteins directly pulled down purified His-p62 in vitro. **C** Schematic representation of the domain architecture of the HECTD3 protein. **D** The DOC domain (216–393 aa) of HECTD3 is sufficient for p62 binding, as determined by the GST pulldown assay. Different GST-fused HECTD3 protein fragments were co-expressed with p62-Flag in HEK293T cells. GST was used as the negative control. **E** Schematic representation of GST-tagged p62 and its truncated mutants. **F** The PB1 domain (1–121 aa) of p62 is required for interactions with HECTD3, as determined by the GST pulldown assay. HEK293T cells were cotransfected with Flag-HECTD3 and GST-p62 or its truncated mutants. **G** HECTD3 ubiquitinates p62 in an E3 ligase activity-dependent manner in vitro. Recombinant purified His-p62 was incubated with UbE1, UbcH5b, HA-Ub, ATP, and HECTD3 or its catalytic inactive mutant C823A. His-p62 was pulled down using the Ni^2+^-NTA beads and ubiquitinated p62 protein was detected using anti-HA Ab. **H** HECTD3 ubiquitinates p62 at K420. HEK293T cells were cotransfected with HECTD3 or HECTD3-C823A and p62-Flag or p62-K420R-Flag for 24 h. The cell lysates were subjected to immunoprecipitation using the anti-Flag M2 beads under a denaturing condition, followed by WB using the indicated Abs. **I** HECTD3 ubiquitinates p62 with K29 and K-63 linked polyubiquitin chains. HECTD3, p62-Flag and HA-Ub (WT; K0; K63 only; K63R; K29 only; or K29R mutants) were expressed in HEK293T cells as indicated. The ubiquitinated p62 was immunoprecipitated using the anti-Flag M2 beads and probed with anti-HA Ab.

Next, we investigated the mechanisms by which HECTD3 promotes DDR. We previously identified that UbcH5b serve as a predominant E2 for HECTD3 [30]. It has been reported that UbcH5b directly interacts with and promotes p62 ubiquitination [34] and we found that PC3-15 inhibits UbcH5b-HECTD3 mediated p62 ubiquitination [30]. More importantly, p62 has been reported to inhibit DDR [20]. Therefore, we wondered whether HECTD3 is an E3 ubiquitin ligase for p62. To test this hypothesis, we first performed co-immunoprecipitation (Co-IP) experiments and found that endogenous p62 pulled down endogenous UbcH5b and exogenously expressed Flag-HECTD3 (Fig. 4A). In addition, purified recombinant GST-HECTD3 and GST-UbcH5b specifically pulled down purified His-p62, indicating that HECTD3 and UbcH5b directly interacted with p62 (Fig. 4B).

To determine which domain of HECTD3 was responsible for its interaction with p62, we performed GST pull-down experiments using a series of truncated HECTD3 GST-fusion proteins (Fig. 4C) and p62-Flag. The DOC domain (216–393) of HECTD3 was responsible for p62 interactions (Fig. 4D). Furthermore, we generated a series of p62 truncation mutants (Fig. 4E) and performed GST pull-down assays to demonstrate that p62 interacts with Flag-HECTD3 through the N-terminal PB1 domain (Fig. 4F). Taken together, these results suggested that the DOC domain of HECTD3 interacts with the PB1 domain of p62.

Next, we examined whether HECTD3 ubiquitinates p62 in vitro. We assembled an in vitro ubiquitination system containing ATP, E1 Ub-activating enzyme (UbE1), UbcH5b, His-p62, HECTD3, and HECTD3-C823A (a catalytically inactive mutant). As shown in Fig. 4G, HECTD3 promotes p62 ubiquitination in an E3 ligase activity-dependent manner. It has been reported that UbcH5b mediates p62 ubiquitination at K420 [34]. To test whether HECTD3 also ubiquitinates p62 at this site, we mutated K420 to R and performed a ubiquitination assay in HEK293T cells. As expected, p62-K420R was not ubiquitinated by HECTD3 in HEK293T cells (Fig. 4H and S3A), suggesting that HECTD3-mediated p62 ubiquitination occurred at K420.

p62 has been reported to be ubiquitinated with K29-, K33-, K48- and K63-linked polyubiquitin chains [34–38]. Next, we examined the association between HECTD3 and p62 poly-ubiquitination. We transfected various HA-Ub mutants into HEK293T cells and found that HECTD3-mediated p62 ubiquitination was primarily mediated by K63 and K29 because WT, K63 only, and K29 only Ub efficiently supported HECTD3-mediated p62 ubiquitination (Fig. 4I and S3B). In addition, K63R and K29R Ub did not support HECTD3-mediated p62 ubiquitination (Fig. 4I). Thus, we conclude that HECTD3 likely promotes p62 ubiquitination at K420 with K63 and K29 linked polyubiquitin chains.

### HECTD3 positively regulates RNF168 activity by promoting autophagy

**Fig. 5:**
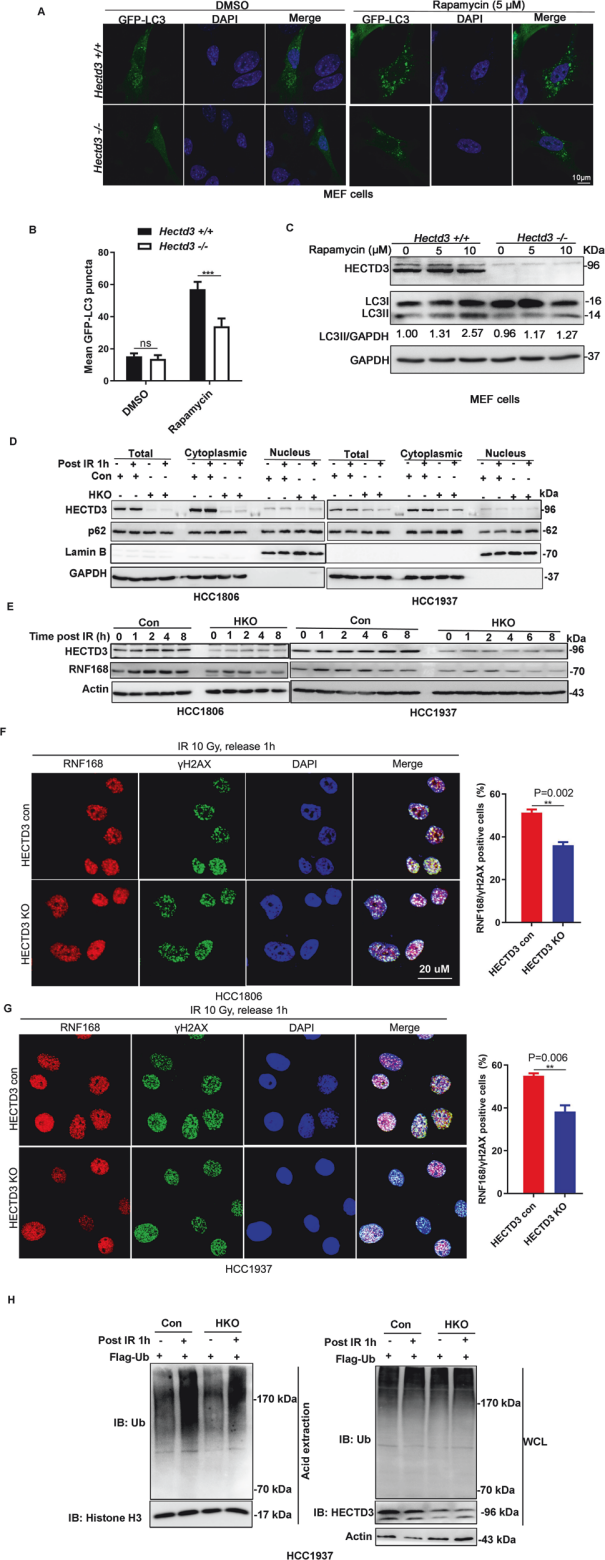
HECTD3 positively regulates RNF168 activity by promoting autophagy. **A** Hectd3 depletion decreased rapamycin induced autophagy. Hectd3 WT and KO MEF cells were treated with 5–10 μM rapamycin for 4 h. The protein levels of LC3-II were measured by WB. **B** Quantitation of GFP-LC3 puncta in Hectd3 + /+ and Hectd3 -/- MEFs treated with rapamycin. All data are represented as the mean ± SEM. *p < 0.05. Student’s t test was used for the statistical analysis. **C** Hectd3 knockout decreased rapamycin-induced autophagy. GFP-LC3 was transfected into MEF cells for 48 h. The cells were treated with rapamycin (5 μM) for 4 h. Puncta formation indicated autophagy. **D** HECTD3 knockout leads to p62 accumulation in nucleus after IR. HECTD3 (Con) and HECTD3 (KO) HCC1806 and HCC1937 cells were irradiated with 10 Gy and released for 1 h. WB was performed to determine p62 protein levels in the nucleus and cytoplasm. **E** HECTD3 knockout decreased RNF168 protein levels. HECTD3 (Con) and HECTD3 (KO) HCC1806 and HCC1937 cells were irradiated with 10 Gy and RNF168 was detected by WB. **F**, **G** HECTD3 knockdown reduced IR-induced RNF168 foci after IR treatment in HCC1806 and HCC1927 cells. Cells transfected with HECTD3Con or HECTD3 KO were treated with IR (10 Gy) after 1 h, the cells were immunostained with anti-RNF168 (red), anti-γH2AX (green) and DAPI (blue) and analyzed using a confocal microscopy. The cells containing > 5 RNF168 foci were recorded as RNF168/γH2AX positive cells, whose percentage was averaged from at least 100 cells. The data are displayed as mean plus SD of three counts, and statistical significance was calculated and represented as the P-value. *p < 0.05 and ns, not significant. Scale bars, 20 μm. **H** HECTD3 increases histones ubiquitination upon IR. HECTD3 (Con) and HECTD3 (KO) HCC1937 cells were stably expressed Flag-Ub, then were irradiated with 10 Gy and subjected to acid extraction of chromatin 1 h after IR.

It has been reported that p62 ubiquitination at K420 enhances its autophagy receptor function [34, 39]. Therefore, we wonder whether HECTD3 promotes autophagy, we first transfected the GFP-LC3 plasmid into *Hectd3* WT/KO MEF and then treated them with rapamycin, a well-known mTOR inhibitor, and an autophagy inducer. We observed that GFP-LC3 puncta formation was significantly reduced in *Hectd3* KO MEF cells (Fig. 5A, B). Consistent with this, LC3-II protein levels decreased in Hectd3 KO MEF (Fig. 5C). Next, we examined autophagy induction in the HCC1806 and HCC1937 cells. As expected, LC3-II protein levels decreased upon rapamycin treatment, while BafA1 treatment led to LC3-II accumulation in HECTD3 KO TNBC cells (Figure S4). Consistently, we observed that HECTD3 KO decreased IR-induced autophagy due to a decrease in LC3 II protein levels and increased p62 accumulation in the nucleus (Fig. 5D and S5). In addition, several reports have indicated the RNF168 protein level was decreased in autophagy-deficient cells following IR treatment [40]. As shown in Fig. 5E, as we expected, the RNF168 protein levels were increased in response to IR. Interestingly, we also observed that PC3-15 had increased nuclear p62 protein levels and reduced RNF168 protein levels in response to IR (Figures S6A, B).

It has been reported that nuclear p62 directly interacted with RNF168 and inhibits RNF168 activity [20]. We next tested whether p62 affects RNF168 recruitment to DSB sites, we found the IR-induced RNF168 foci formation (Fig. 5F, G) was suppressed in HECTD3 KO cell lines. Consistently, IR-induced histone ubiquitination (Fig. 5H) was decreased in HECTD3 KO cells. Collectively, these data showed that HECTD3 deficiency led to nuclear p62 accumulation and inhibited RNF168 mediated DNA damage repair signaling.

### HECTD3 KD sensitizes TNBC cells to IR depending on p62

**Fig. 6:**
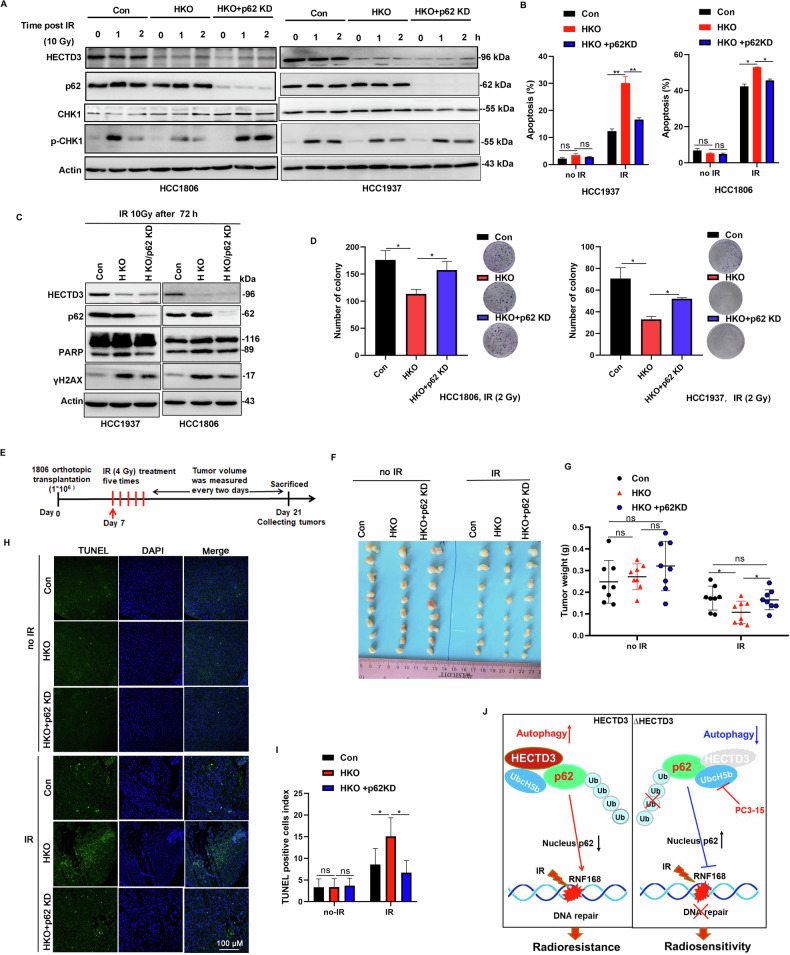
HECTD3 mediated IR resistance through p62. **A** HECTD3 KO-impaired pCHK1 (S345) was reversed by p62 knockdown. HECTD3 (Con), HECTD3 (KO) and p62 knockdown/HECTD3 KO HCC1806 and HCC1937 cells were irradiated with 10 Gy. CHK1 and pCHK1 were detected by WB. **B** p62 knockdown restored IR resistance in HECTD3 KO HCC1806 and HCC1937 cells. Apoptosis were analyzed by flow cytometry after annexin V/PI staining at 72 h post-IR. Data are mean ± SD. Statistical analysis was performed using two-tailed unpaired t-tests. Data are representative of three independent experiments. *p < 0.05 and **p < 0.01; ns, not significant. **C** p62 knockdown decreased the accumulation of ${\rm{\gamma }}$H2AX and PARP cleavage in HECTD3 KO HCC1806 and HCC1937 cells. Cells were treated with IR (10 Gy) and harvested for WB at 72 h post-IR. **D** p62 knockdown restored IR resistance in HECTD3 KO HCC1806 and HCC1937 cells. Clonogenic formation of HCC1806 and HCC1937 cells in response to IR were measured. Data are mean ± SD. Statistical analysis was performed using two-tailed unpaired t-tests. Data are representative of three independent experiments. *p < 0.05 and n.s, not significant. **E** Schematic diagram of radiotherapy process for HECTD3 Con, HECTD3 KO and HECTD3 KO/p62 KD tumors. **F**–**I** HECTD3 KO increased the efficacy of radiotherapy and p62 KD rescues HECTD3 KO mediated IR sensitivity. Mice with HECTD3 Con, HECTD3 KO and HECTD3 KO/p62 KD tumors were divided into two groups, control or radiotherapy (*n* = 4 per group). 21 days post five times radiotherapy (4 Gy/time), the mice were euthanized. The image of tumors was shown (**F**); the tumor weight (**G**) and TUNEL positive ratio (**H**–**I**) were recorded. Data are mean ± SD. Statistical analysis was performed using two-tailed unpaired t-tests. *p < 0.05 and ns, not significant. Scale bar, 100 μm. **J** The working model of this study.

To further validate our hypothesis that HECTD3 KO sensitizes TNBC cells to IR due to p62 nuclear accumulation, we silenced p62 expression in HECTD3 KO cells. We observed that pCHK1 levels were significantly reduced in HECTD3 KO cells. Importantly, this reduction was rescued by p62 deletion, indicating that HECTD3 promotes HR activation in a p62-dependent manner (Fig. 6A). Furthermore, p62 KD markedly decreased apoptosis in HECTD3 KO cells 72 h post-IR, as measured by annexin V staining (Fig. 6B) and PRAP cleavage (Fig. 6C). Consistently, p62 silencing significantly decreased the levels of γH2AX (Fig. 6C) and partly restored the colony formation (Fig. 6D). These data indicated that p62 KD rescued HECTD3 KO mediated IR sensitization in vitro.

Furthermore, we tested whether this conclusion stands in vivo using three groups (Control, HECTD3 KO, and HECTD3 KO/p62 KD) of HCC1806 cells injected subcutaneously into nude mice. The tumors formed within a week. After low-dose RT or mock therapy, the mice were euthanized and the tumors were dissected to examine their weight and apoptosis (Fig. 6E). Upon IR, we found that the average weight of HECTD3 KO tumors was significantly lower than that of control tumors. p62 KD rescued the tumor weight loss caused by HECTD3 KO (Fig. 6F, G). In addition, there were no differences in terms of mouse body weight among the three groups (Figure S7). Consistently, compared with the other two groups, HECTD3 KO tumors had the highest proportion of TUNEL-positive apoptotic cells after IR (Fig. 6H, I). These results suggest that HECTD3 KO increases the efficacy of RT depending on p62 in vivo.

## Discussion

HECTD3 is a pro-survival E3 ubiquitin ligase in cancers [22]. In this study, we demonstrated that HECTD3 promoted TNBC survival from IR. HECTD3 ubiquitinates p62 at K420 via K63 and K29 linked polyubiquitin chains and promotes autophagy and DSB repair, and its genetic ablation or pharmacological inhibition has shown to be a promising strategy to increase radiosensitivity of cancer cells.

Nuclear p62 directly binds to RNF168 and inhibits RNF168 mediated histone H2A ubiquitination and DNA damage responses, thus increasing the sensitivity of tumor cells to radiation [20]. Wang et al. identified a 1,2,3-triazole derivative of quinazoline 5a (compound 5a) that directly binds to both p62 and RNF168 and promotes their interaction. Compound 5a decreased RNF168-mediated H2A ubiquitination and compromised HR, thereby increasing the sensitivity of HCT-116 cells to IR [41]. Consistently, our results suggest that PC3-15 increases nuclear p62 levels and inhibits RNF168 accumulation upon IR treatment, and PC3-15 increased the antitumor efficacy of IR. Nonetheless, we cannot exclude the possibility that PC3-15’s potential off-target effects.

p62 is a multifunctional scaffold protein involved in various cellular processes, including autophagy, apoptosis, stemness, and DDR [42]. Aberrant p62 expression has been implicated in tumor development and therapeutic resistance [43]. The function of p62 is tightly regulated by ubiquitination. p62 ubiquitination by Keap1/cul3 at K420 to enhances the sequestering activity of p62 and autophagic degradation [39]. UbcH5b/c promotes p62 ubiquitination at K420 to activate the autophagy receptor function under ubiquitin stress [34]. However, SPOP mediates the nondegradative ubiquitination of p62, thereby suppressing autophagy [44]. TRIM44 mediates p62 deubiquitination, leading to oligomerization and nuclear export upon IR, thus promoting DDR [45]. In this study, we demonstrated that HECTD3 mediates p62 ubiquitination at K420 via K63 and K29 linked polyubiquitin chains, resulting in the activation of autophagy. However, we did not find that HECTD3 regulates p62 oligomerization (data not shown).

Accumulating evidence indicates that tumors with elevated RNF168 levels are more resistant to chemotherapy and RT [46]. Downregulation of RNF168 protein expression sensitizes cancer cells, particularly BRCA1-deficient cells, which are resistant to DNA damage agents [47]. RNF168 is considered the rate-limiting determinant for the recruitment of key DDR proteins to damaged chromatin. Thus, RNF168 levels were tightly controlled. It has been reported that TRIP12 and UBR5 are the two major E3 ligases responsible for degrading RNF168 [48]. USP14 negatively regulates RNF168 protein expression in response to IR [40]. In addition, the mTORC1-S6K pathway regulates DDR via the phosphorylation of RNF168 at S60, which inhibits its E3 ligase activity. Depletion of the tumor suppressor LKB1 activates mTORC1 and decreases RNF168 abundance, subsequently impairing DDR [49]. Interestingly, HECTD3 mediated LKB1 ubiquitination promotes radiation resistance in glioma [27]. Recently, Li et al. reported that IRAK1 promotes radio resistance of glioma cells by suppressing autophagic cell death via decreasing the HECTD3 mediated ubiquitination and degradation of PRDX1 [50]. In this study, we demonstrated that HECTD3 positively regulates RNF168 expression upon IR. HECTD3 ubiquitinates p62 and promotes autophagic degradation. Decreased nuclear p62 levels lead to the activation of RNF168 and DDR. We cannot exclude the possibility that HECTD3 sustains RNF168 expression upon IR treatment via LKB1 in TNBC cells.

In summary, our study revealed a previously unexplored connection between HECTD3 and two critical pathways of the tumor cell response to RT: DDR and autophagy. HECTD3 positively regulates DDR and autophagy and promotes radio-resistance in a p62 dependent manner. PC3-15 increases nuclear p62 accumulation and inhibits RNF168; therefore, PC3-15 could be developed as a potential therapeutic agent for sensitizing TNBC cells to RT.

## Materials and methods

### Plasmids

All plasmids used in this study are listed in Tables S1-2.

### Antibodies and regents

Antibodies against p62/SQSTM1 (ab101266), UbcH5b (ab66602), and γH2AX (ab229914) were purchased from Abcam (Cambridge, UK). Antibodies against LC3B (3868 s), -tubulin (#15115), p-CHK1 (#2348), and PARP (#9582) were purchased from Cell Signaling Technology (Danvers, MA, USA). Antibodies against GAPDH (sc-32233), β-actin (sc-8432), HA (sc-7392), and RNF168 (sc-101125) were obtained from Santa Cruz Biotechnology (Dallas, TX, USA). The FLAG antibody (F1804) was purchased from Sigma-Aldrich (Darmstadt, Germany). The Ub antibody against FK2 (04-263) was obtained from Millipore (Darmstadt, Germany). Lamin B1 (A1910) and CHK1 (A7653) were obtained from AbClonal (Wuhan, CHN). The HECTD3 antibody was described in our previous study [23]. Rapamycin (HY-50898) was obtained from MCE (Shanghai, China).

### Cells and transfection

Human TNBC cell lines MDA-MB-231, HCC1937 and HCC1806 were purchased from Kunming Cell Bank (Chinese Academy of Science, Kunming, China). HCC1806 and HCC1937 were cultured in RPMI 1640 basic (Gibco Life Technologies, Carlsbad, CA) with 5% FBS. MDA-MB-231 were cultured in DMEM/F12 basic (Gibco Life Technologies, Carlsbad, CA) with 5% FBS. HEK293T and MEF cells were cultured in DMEM basic (Gibco Life Technologies, Carlsbad, CA) with 5% FBS. MC1 cells were cultured in DMEM basic (Gibco Life Technologies, Carlsbad, CA) with 10% FBS.

All transfections of plasmids and siRNAs were performed using Lipofectamine 2000 (Invitrogen, Carlsbad, CA, USA) according to the manufacturer’s instructions. All chemically synthesized siRNAs were purchased from RiboBio (Guangzhou, China) and transfected at a final concentration of 20 nM. The shRNA target sequence for the human *p62* gene is 5’-GGAGTCGGATAACTGTTCAAT-3.’ The siRNA target sequences for the human *HECTD3* gene are 5’-GCGGGAACUAGGGUUGAAUTT-3’ and 5’-GGUAUUUCACCUCUUAAGATT-3.’

### Generation of HECTD3 KO breast cancer cell lines

The plasmids for HECTD3 knockout were constructed using the CRISPR-Cas9 system as described previously [32]. The 20 bp guide sequence (5’-GCTGGCTTTCGTGCCGCGAG-3’) was selected from a database of predicted high-specificity protospacer adjacent motif (PAM) target sites ((http://tools.genome-engineering.org). Two complementary oligos containing the HECTD3 sequence and BsmBI (NEB) ligation adapters were synthesized. The annealed oligonucleotide was ligated into the BsmBI-digested lentiCRISPRv2 vector. The sequences of the constructed plasmids were verified using DNA sequencing.

The lentiCRISPRv2 or lentiCRISPRv2-gRNA constructed plasmids combined with psPAX2 and pMD2.G were introduced into HEK293T cells by transfection with Lipofectamine 2000 for 48 h to generate the lentivirus. Lentivirus-infected HCC1806 and HCC1937 cells were selected with 10 μg/ml puromycin (Sigma-Aldrich) for 36 h. Surviving cells were collected to test the knockout effect by WB using an anti-HECTD3 antibody.

### Western blotting and immunoprecipitation

Western blotting and immunoprecipitation methods were described in our previous study [51]. For WB analysis, the samples were mixed with 1 × SDS buffer (60 mM Tris-HCl [pH 6.8], 1% [w/v] SDS, 5% [v/v] glycerol, 0.005% [w/v] bromophenol blue, and 1% [v/v] 2-mercaptoethanol) for 10 min, separated by SDS-PAGE, and transferred to PVDF membranes (Millipore, Billerica). After blocking with 5% nonfat milk in phosphate-buffered saline (Lufei, China) containing 0.1% Tween 20 (A100777; Sangon Biotech, Shanghai, China) the membranes were incubated with the indicated antibodies overnight at 4 °C, followed by incubation with a horseradish-peroxidase-conjugated secondary antibody for 1 h at room temperature. Protein bands were detected with Super ECL Plus (UE, S6009), an ImageQuant LAS4000 (GE, Germany). The p62 rabbit antibody (3 μg) was used to immunoprecipitate the endogenous UbcH5b protein or exogenously expressed Flag-HECTD3 from HEK293T cells. Rabbit IgG (Proteintech, B900610) was used as the negative control. Whole HEK293T lysates in IP buffer composed of 0.5% (v/v) NP-40, 50 mM Tris-HCl (pH 7.4), 50 mM EDTA, 150 mM NaCl, and protease/phosphatase inhibitor cocktails (Biotool, USA) were collected and incubated with Protein A/G (Santa Cruz, sc-2003) for 12 h at 4 °C, followed by washing five times with IP buffer. The immunoprecipitated components were boiled in 2 × SDS loading buffer for 10 min and subjected to WB analysis.

### GST pull-down assays

GST pull-down experiments in HEK293T cells have been described previously [51]. GST-p62/GST-HECTD3 and their truncated mutants were cotransfected with Flag-HECTD3 or p62-Flag in HEK293T for 24 h. HEK293T cell lysates (500 μL) were incubated with Glutathione Sepharose beads (20 μL for each sample) for 2 h at 4 °C on a rotating platform, and the beads were washed five times and resuspended in ice-cold lysis buffer. The samples were subjected to WB by blotting with the indicated antibodies. Similarly, purified His-p62 (3 μg) was mixed with GST-UbcH5b (6 μg) or GST-HECTD3 (6 μg) and incubated with Glutathione Sepharose beads (20 μL for each sample) for 2 h at 4 °C on a rotating platform; the beads were washed five times and resuspended in ice-cold lysis buffer. The samples were subjected to WB blotting using an anti-p62 antibody.

### Immunofluorescence and confocal microscopy

MEF cell lines were transfected with expression plasmids for the fluorescent fusion protein GFP-LC3 and subjected to fluorescence microscopy analyses. The cells were fixed with 4% (w/v) paraformaldehyde for 30 min. The cell nuclei were counterstained with DAPI (HY-D0814, MCE, Shanghai, China). Fluorescence microscopy was performed using a Carl Zeiss LSM880 microscope (Germany). Puncta formation by GFP-LC3 was quantified as follows: 100 cells were assessed from three fields in three independent experiments.

For γH2AX and RNF168 foci experiments, HCC1806 or HCC1937 cells were treated with/without IR, fixed with 4% paraformaldehyde for 15 min at room temperature and then incubated overnight at 4 °C with mouse anti-γH2AX (Millipore) and mouse anti-RNF168 antibodies. Labelling was performed using Alexa Fluor 555-labelled goat-anti-rabbit immunoglobulin secondary antibodies (Molecular Probes). Cells were stained with DAPI for 5 min and mounted using Mowiol Mounting Solution (Calbiochem). The slides were examined under a Carl Zeiss LSM880 microscope (Germany). Images were acquired at 100 × magnification using Leica Image Manager software.

### Ubiquitination assays

Ubiquitination assays were performed as described before [23]. For the in vitro ubiquitination assays, bacteria expressed human HA-Ubiquitin (HA-Ub, 0.4 μg), Ub activating enzyme (UbE1, 0.4 μg)), Ub-conjugating enzyme (UbcH5b, 0.54 μg), His-p62 (5 μg), GST-HECTD3 (3 μg), or HECTD3-C823A (3 μg) were added into the reaction buffer to the final volume of 10 μl and incubated at 37 °C for 40 min. Ubiquitinated HECTD3 proteins were detected by WB using an anti-HA antibody. UBE1 (E-305), UbcH5b (E2-622), HA-Ub (U-110), and reaction buffer (B-71) were purchased from Boston Biochem (Boston, MA, USA). For ubiquitination assays, HEK293T cells were transiently transfected with HA-Ub and other plasmids as necessary in six-well plates. Two days after transfection, the cells were harvested in 150 μl of SDS lysis buffer (50 mM Tris-Cl, pH 6.8, 1.5% SDS). The samples were then boiled for 15 min. We diluted 120 μl of protein lysate with 1.2 ml of BSA buffer (50 mM Tris-Cl, pH 6.8, 180 mM NaCl, 0.5% CA630, 0.5% BSA) and incubated it with anti-Flag M2-agarose overnight at 4 °C with rotation. The beads were collected by centrifugation at 10,000 g for 30 s at 4 °C and washed three times with 1 ml of ice-cold BSA buffer. Proteins were resuspended in 50 μl of SDS sample loading buffer and analyzed by WB using the anti-HA antibody.

### Histone acid extraction

Cells were lysed in 1 ml of hypotonic lysis buffer (10 mM Tris-HCl [pH 8.0], 1 mM KCl, 1.5 mM MgCl_2_, 1 mM DTT, and protease inhibitors). Intact nuclei were harvested by centrifugation. Nuclei were resuspended in 400 µl of 0.2 M sulfuric acid (10:910) and incubated for at least 30 min. The supernatant containing histones was harvested, and trichloroacetic acid (the final concentration of TCA was 33%) was added and incubated on ice for 30 min. The histone pellets were harvested, washed with acetone, and dissolved in ddH_2_O.

### COMET assays

The COMET assay has been previously described in detail [52]. Briefly, TNBC cells were treated with/without 2 Gy IR, collected at 8 h post-IR, and embedded in 1% low-gelling-temperature agarose. Embedded cells were lysed overnight and subjected to electrophoresis under alkaline conditions. The slides were stained with propidium iodide and observed under a Carl Zeiss LSM880 microscope. Images were analyzed using the ImageJ plug-in in Open Comet software, and the tail moment was calculated as tail length × tail DNA%. At least 100 cells were examined on each slide and the results were averaged.

### Apoptosis assays

HCC1806 and HCC1937 cells were treated with IR (10 Gy) and collected 72 h post-IR. Apoptotic cells were analyzed with an FITC Annexin V Apoptosis Detection Kit (556547, BD Pharmingen, CA, USA) using an Accuri C6 flow cytometer (BD Biosciences, CA, USA). The apoptosis rate was quantified using BD FACSAria with Cell Quest research software. PARP protein levels were detected by WB. The TUNEL assay was performed as described previously [53]. TUNEL kits were purchased from Roche (11684795910). TUNEL-positive cells were counted using a Carl Zeiss LSM880 microscope (Germany).

### Colony formation assays

Breast cancer cell lines (8000 cells) were seeded on 6 cm dishes, treated with DMSO, IR, or PC3-15, and cultured for an additional 10–14 days. The colonies were stained with crystal violet and counted.

### Mouse experiments

All animal experiments were performed in accordance with the institutional ethical guidelines for animal care and approved by the Animal Resource Center at the Kunming Institute of Zoology (IACUC-PA-2022-03-023).

BALB/c nude mice (6 weeks, female, Animal Co. Ltd., Hunan, China) were selected randomly and orthotopically implanted with 1 × 106 HCC1806 cells in 50% Matrigel (injection volume, 75 μL/mice). When the tumor volume reached approximately 50–70 mm^3^, half of the mice in each group (*n* = 4) received 20 Gy (4 Gy, five times) of IR exposure, and tumor growth and mouse weight were monitored every two days for 21 days. The method for establishing a patient-derived xenograft (PDX) model was based on our previous studies [54]. Briefly, xenografts derived from human breast cancer cells (MC1) were serially propagated in the mammary fat pads of nude mice without in vitro culture. The resulting tumors exhibited characteristics of ER-negative, PR-negative, and HER2-negative invasive ductal carcinoma. To obtain MC1 cells, the tumors were minced and dissociated using F12/DMEM medium supplemented with 5% fetal bovine serum, 300 U/mL collagenase, and 100 U/mL hyaluronidase (Sigma) at 37 °C for 2 h. The resulting cell suspension was centrifuged at 400 g for 5 min, and the pellet was resuspended in red blood cell lysis buffer (eBioscience, San Diego, CA) and incubated at 37 °C for 5 min to remove red blood cells. After centrifugation at 400 g, the pellet was resuspended in F12/DMEM medium supplemented with 10% fetal bovine serum and filtered through a 40 μm nylon mesh (BD Biosciences). 5 × 10^5^ MC1 cells were injected into the fat pads of female nude mice. Mice carrying MC1 xenografts were randomly distributed into four equal groups (*n* = 4) when the tumor size reached approximately 70 mm^3^. The control group was given vehicle alone and without IR and the treatment group received IR (3 Gy × 4) and PC3-15 (50 mg/kg) alone or in combination via intraperitoneal injection every two days. All tumors were measured every three days using a Vernier caliper, and the volume was calculated according to the formula: π/6 × length × width^2^. Measurements and data processing were performed blindly.

### Statistical analysis

All statistical analyses were performed using Prism version 9.0 (GraphPad). Statistical analysis was performed using the two-tailed Student’s *t*-test, unless otherwise noted. *P* < 0.05 was considered to indicate statistical significance. The numbers of cells, mice, and replicates (n) for each experiment are indicated in the figure legends. For bar and line graphs, data are presented as mean ± standard deviation unless specified in the legends. The immunofluorescence micrographs represent three independent experiments with the indicated cells under the same treatment. WB data were obtained from the respective experiments, processed in parallel, and are representative of three independent experiments, unless otherwise specified in the legends.

## Supplementary information

supplemental material

## Data Availability

All analyzed and generated data are presented in the published article and supplementary information. The datasets used and/or analyzed during thecurrent study are available from the corresponding author on reasonable request.
