# Supplementary material for: Targeting the HECTD3-p62 axis increases the radiosensitivity of triple negative breast cancer cells
Source: Cell Death Discov. 2024 Nov 1;10:462. doi: 10.1038/s41420-024-02154-5 (PMC11530666; doi:10.1038/s41420-024-02154-5)
Supplement: Supplementary file 1 — supplemental material [file 41420_2024_2154_MOESM1_ESM.docx]

**Targeting the HECTD3-p62 axis increases the radiosensitivity of triple negative breast cancer cells**

Maobo Huang^#1,2^, Wenjing Liu^#3^, Zhuo Cheng^1,4^, Fubing Li^5^, Yanjie Kong^6^, Chuanyu Yang^1^, Yu Tang^3^, Dewei Jiang^1^, Wenhui Li^3^, Yudie Hu^7^, Jinhui Hu^*7^, PemaTenzin Puno^*8^, Ceshi Chen^*1,3,5^

^1^ Kunming Institute of Zoology, Chinese Academy of Sciences, Kunming, 650201, Yunnan, China.

^2^ The First People's Hospital of Kunming City (The Affiliated Calmette Hospital of Kunming Medical University), Kunming, 650224, China.

^3^ The Third Affiliated Hospital, Kunming Medical University, Kunming, 650118, China.

^4^ Kunming College of Life Sciences, University of the Chinese Academy of Sciences, Kunming, 650204, China.

^5^ Academy of Biomedical Engineering, Kunming Medical University, Kunming, 650500, China.

^6^ Biobank, Shenzhen Second People's Hospital/ the First Affiliated Hospital of Shenzhen University Health Science Center, Shenzhen, 518035, China.

^7^ The First Hospital of Hunan University of Chinese Medicine, Changsha, 410007, Hunan, China.

^8^ State Key Laboratory of Phytochemistry and Plant Resources in West China, Kunming Institute of Botany, Chinese Academy of Sciences, Kunming, 650201, Yunnan, China.

**# These authors contribute equally.**

***Correspondence authors**

**Supplementary Tables**

**Table S1.** Primers used in this study for cloning

| Target genes | Primer sequences |
| --- | --- |
| His-p62 | F: 5'CACCATGGCCATGTCCTACGTGAAGGATG 3' R: 5'TTA TCACAACGGCGGGGATGCTTTGAATA 3 |
| p62K420R-Flag | F: 5' CTGCAGACCAGGAACTATGACATCGGAGCGGCT 3' R: 5' GTCATAGTTCCTGGTCTGCAGGAGCCTGGTGAG 3' |
| GST-p62 | F: 5'GATCTGGTTCCGCGTGGATCCATGGCGTCGCTCACCGTG 3' R: 5'CCTCACTCTAGAGTCGCGGCCGCTCACAACGGCGGGGGATG 3' |
| GST-p62∆1-121 | F: 5' CGCGAGATTGCCATGTCCTACGTGAAGGATGACA 3' R: 5' GGACATGGCCTCGCGCGCCGCGTCCTCCTTGCCCA 3' |
| GST-p62∆122-168 | F: 5' CGCAACATGCCCAGCCCCTTCGGGCACCTGTCTG 3' R: 5' GGGGCTGGGCACCATGTTGCGGGGCGCCTCCTGAG 3' |
| GST-p62∆220-253 | F: 5' GCTTCTGGTGAAGTTGATATCGATGTGGAG 3' R: 5' ATCAACTTCACCAGAAGCTGATTCTGCCGT 3' |
| GST-p62∆320-340 | F: 5' GAGTCCGAGTCAAAAGAAGTGGACCCGTCTAC 3' R: 5' TTCTTTTGACTCGGACTCCAAGGCGATCTTCCT 3' |
| GST-p62∆343-440 | F: 5' ATCTGGTTCCGCGTGGATCCTCAAAAGAAGTGGACCCGTCTAC 3' R: 5' CACTCTAGAGTCGCGGCCGCTCACAACGGCGGGGGATGCTTTG 3' |

**Table S2.** Plasmids used in this study

| plasmid | vector | Ref. |
| --- | --- | --- |
| GST-HECTD3 | pGEX-6p-1 | [1, 2] |
| GST-H1-215 | PEBG | [1] |
| GST-H216-393 | PEBG | [1] |
| GST-H109-393 | PEBG | [1] |
| GST-394-510 | PEBG | [1] |
| GST-512-861 | PEBG | [1] |
| HECTD3 | PCDA 3.1 | [1, 2] |
| HECTD3C823A | PCDA 3.1 | [1, 2] |
| HECTD3 sg1# | lentiCRISPRv2 |  |
| GST-UbcH5b | pGEX-6p-1(gift from Prof. Hu) | [4] |
| p62-Flag | PCDH (gift from Prof. Hu) | [4] |
| His-p62 | pET-28a |  |
| p62K420R-Flag | PCDH (gift from Prof. Liu) | [5] |
| GST-p62 | PEBG |  |
| GST-p62∆1-121 | PEBG |  |
| GST-p62∆122-168 | PEBG |  |
| GST-p62∆220-253 | PEBG |  |
| GST-p62∆320-340 | PEBG |  |
| GST-p62∆343-440 | PEBG |  |
| GFP-LC3 | PEGF ( gift from Prof. Yao) | [6] |
| Flag-Ub | PCDH | [9] |
| HA-Ub | pCDNA3 | [7] |
| HA-Ub K0 | pCDNA3 | [8] |
| HA-Ub K63 | pCDNA3 | [1] |
| HA-Ub K63R | pCDNA3 | [1] |
| HA-Ub K48 | pCDNA3 | [1] |
| HA-Ub K33 | pCDNA3 | [8] |
| HA-Ub K29 | pCDNA3 | [8] |
| HA-Ub K29R | pCDNA3 | [8] |
| HA-Ub K27 | pCDNA3 | [8] |
| HA-Ub K11 | pCDNA3 | [8] |
| HA-Ub K6 | pCDNA3 | [8] |

**Supplementary Figures**

**
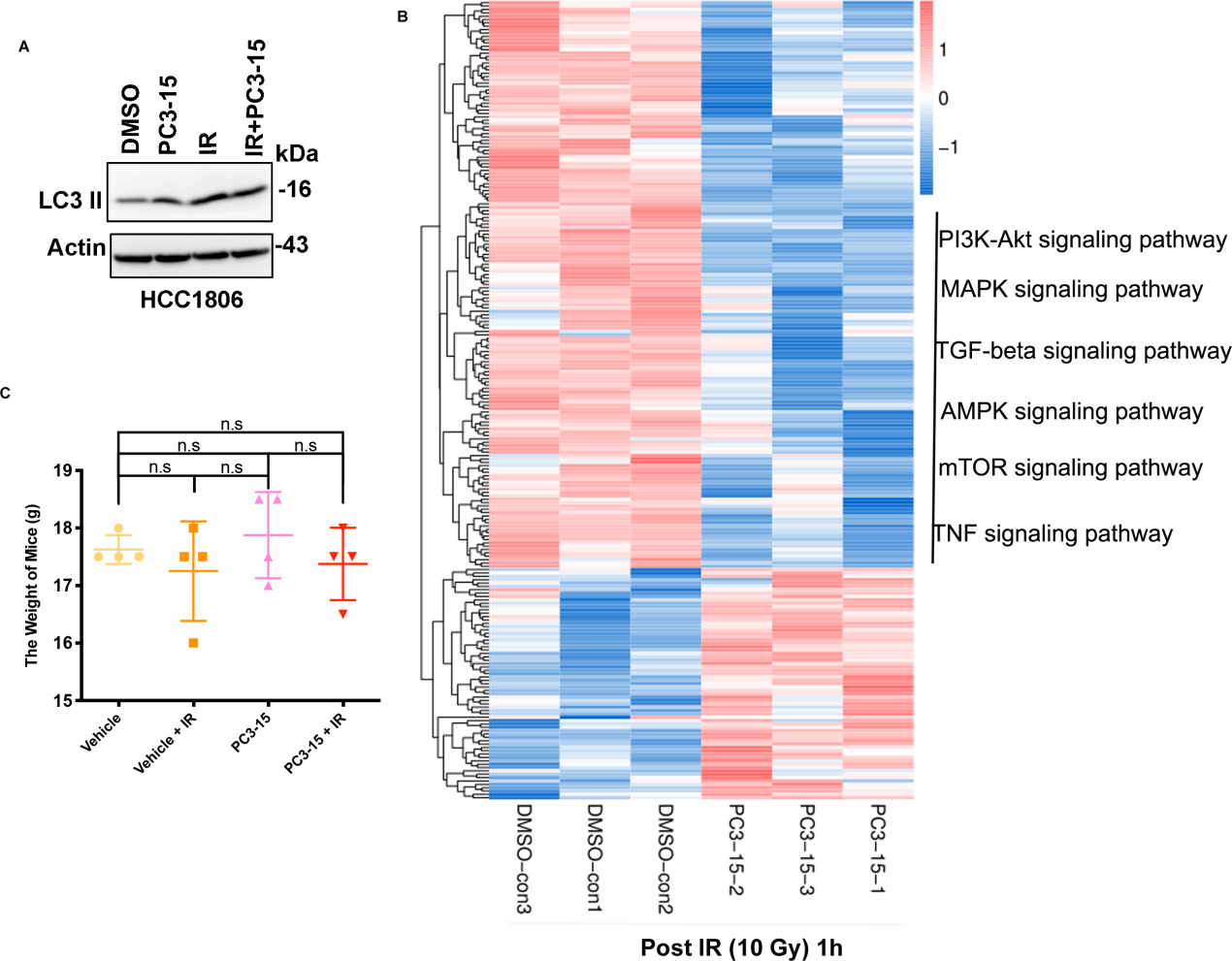
**

**Figure S1. PC3-15 increases radiosensitivity of TNBC cells by inhibiting DDR**

**A.** PC3-15 inhibited IR indcced autophagy. HCC1806 cells were treated with PC3-15 (20 μM) and IR (10 Gy) alone or in combination, and harvested at 1 h post-IR for WB to measure LC3 II protein levels.

**B.** RNA-seq analysis revealed PC3-15 inhibited IR-induced autophagy ralated signaling pathway in HCC1806 cells.

**C.** There was no significant difference in mouse weight among groups at 12 day.


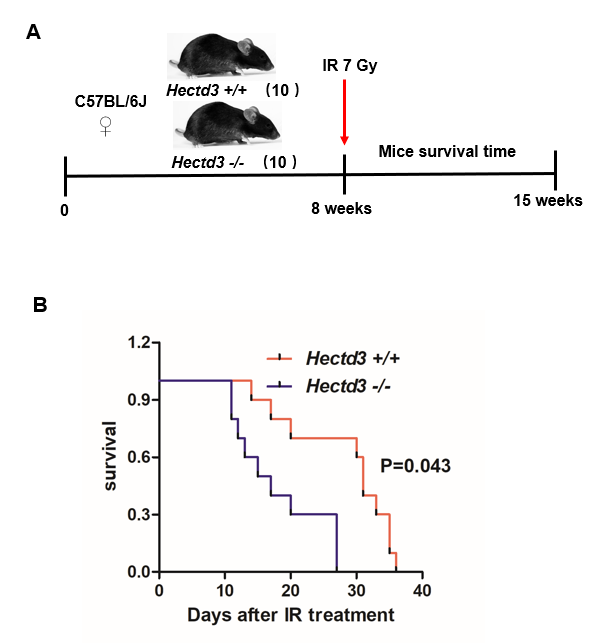


**Figure S2. Hectd3 promotes mouse survival from irradiation.**

1. Schematic diagram for the constructions of whole-body irradiation of Hectd3 KO mice.
2. The survival time of *Hectd3* knockout mice became shorter after irradiation. Kaplan-Meier survival curves of irradiated *Hectd3* WT versus KO male C57Bl/6 mice (n = 10 per genotype). Statistical analysis was performed using the log-rank test.


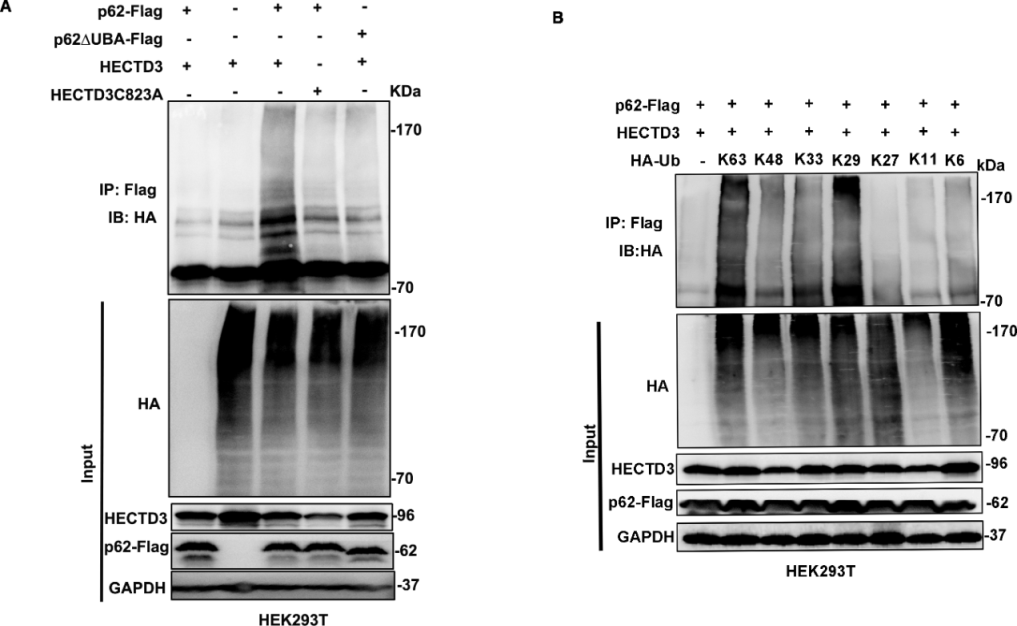


**Figure S3. HECTD3 ubiquitinates p62 at UBA domain with K29 and K63 linked polyubiquitin chains**

**A.** HECTD3 ubiquitinates p62 at UBA domain. HEK293T cells were co-transfected with HECTD3 or HECTD3-C823A and p62-Flag or p62∆UBA-Flag for 24h. The cell lysates were subjected to immunoprecipitation using the anti-Flag M2 beads under a denaturing condition, followed by WB using indicated Abs.

**B.** HECTD3 ubiquitinates p62 with K29- and K63-linked polyubiquitin chains. HECTD3, p62-Flag and K only HA-Ub were expressed in HEK293T cells as indicated. The ubiquitinated p62 was immunoprecipitated using the anti-Flag M2 beads and probed with anti-HA Ab.

**
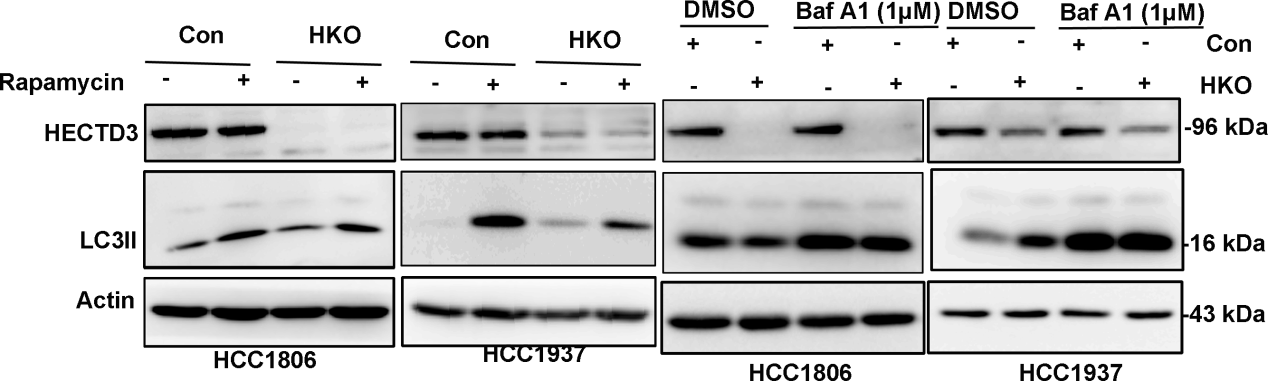
**

**Figure S4.** HECTD3 promotes autophagy in HCC1806 and HCC1937 cells. HECTD3 KO decreased LC3II protein levels upon rapamycin treatment, while increased LC3II protein levels upon Bafilomycin A1 treatment. HECTD3 (Con) and (KO) HCC1806 and HCC1937 cells were treated with 5 μM rapamycin or 1 μM Baf A1 for 4 h. The protein levels of LC3-II were measured by WB.


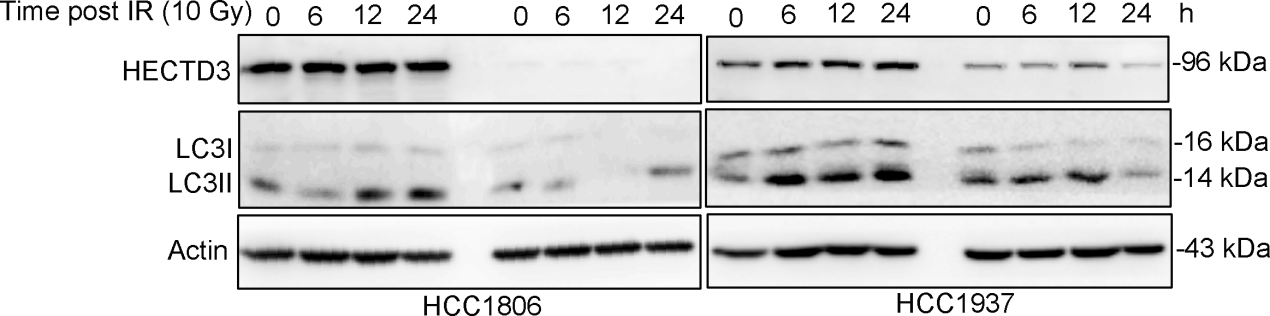


**Figure S5. HECTD3 promotes IR induced autophagy in TNBC cells**. HECTD3 (Con) and HECTD3 (KO) HCC1806 and HCC1937 cells were irradiated with 10 Gy and released for 6, 12, and 24 h. WB was then performed to detect LC3II protein levels.


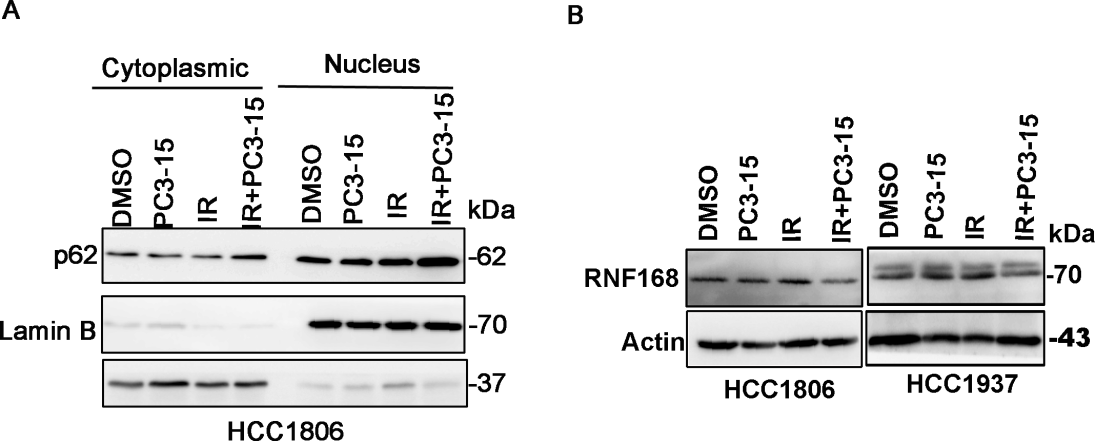


**Figure S6. PC3-15 promotes nuclear p62 accumulation and reduced RNF168 protein levels in TNBC cells**.

**A.** PC3-15 increased nuclear p62 accumulation upon IR. HCC1806 cells were treated with PC3-15 (20 μM) and IR (10 Gy) alone or in combination, and harvested for WB at 4 h post-IR.

**B.** PC3-15 downregulated RNF168 protein levels upon IR. HCC1806 and HCC1937 cells were treated with PC3-15 (20 μM) and IR (10 Gy) alone or in combination, and harvested for WB at 1 h post-IR.

**
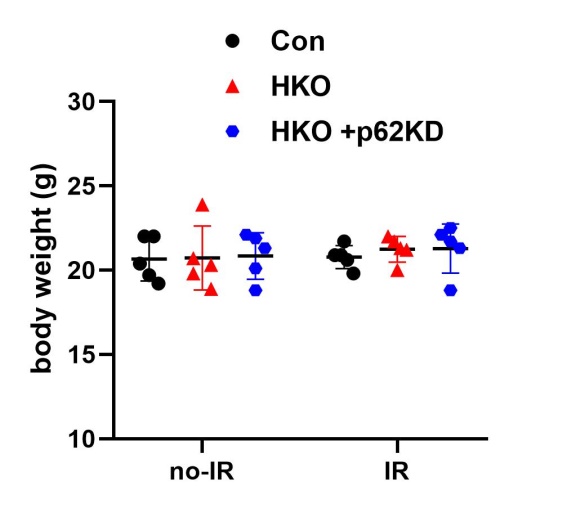
**

**Figure S7.**  IR treatment did not significantly affect the HCC1806 xenograft mouse body weights. Mouse body weight was measured at the sacrificed day.

**Supplementary methods**

**Protein purification**

The GST-tag protein (GST-HECTD3, GST-UbcH5b) purification was described in our previous study [1]. The expression and purification of the His-p62 was conducted according to a previous study [47]. For protein expression, *Escherichia coli* strain Rosetta 2 (DE3) (TSC04, TsingKe, Kunming, China) bearing the expression plasmids were grown at 37 °C to 0.6 OD_600_, then induced with 0.6 mM isopropyl-β-D-thiogalactopyranoside (IPTG, A100487, Sangon Biotech, Shanghai, China) at 16 °C for 10 h, and harvested by centrifugation. After sonication of the bacteria, soluble GST fusion proteins were affinity purified through a Glutathione Sepharose 4B column (G4510, Sigma, Germany) and His tag proteins were purified by Ni^2+^-NTA beads (#44325, MCE, Shanghai, China). These proteins were identified by SDS-PAGE using Coomassie blue staining.

**Isolation and culture of MEF cells**

We isolate MEF cells according to the standard protocols with modifications [10]. BALB/c mouse embryos at 12.5 to 14.5 days of gestation were used for isolation of MEFs. Briefly, mice were sacrificed by cervical dislocation and soaked with 70% ethanol solution. After sterilization with ethanol, the abdomen was cut and the uterine horn was gently removed. Then, the uterine horn was rinsed in the 50 ml falcon tube containing PBS without Ca^2+^ and Mg^2+^ (PBS-CM) (Sigma-Aldrich, Germany). Under sterile condition, the uterine horn was placed on the sterile petri dish. After yolk sac and placenta removal by a clean cutter, the tissue was washed twice with PBS-CM to exclude blood before transferring to another sterile petri dish. A total of 12 embryos were harvested from WT or KO mice. The embryos tails, heads and internal red organs (heart and liver) were discarded and the rest of fetus tissues were minced finely into the smallest pieces with a sterile blade. The resulting tissue slurries were transferred into two 50 ml canonical tubes and incubated for 20 min or 40 min at 37 °C with 5 ml of 0.25% trypsin/EDTA, and 100 Kunitz units of DNase I per each embryo. The tip of the P1000 pipet was cut, and the tissues were gently pipetted every 10 min to dissociate clumpy tissue. At the end of the incubation period, clumpy tissue was removed, and trypsin activity was neutralized with 5 ml FBS. To separate the cells, the suspension was centrifuged at 500 g for 5 min. The supernatant was discarded and the cell pellet was re-suspended in MEF media. MEF cells were cultured in DMEM/F12 basic (Gibco Life Technologies, Carlsbad, CA) with 10% FBS.

**Original werstern blot**


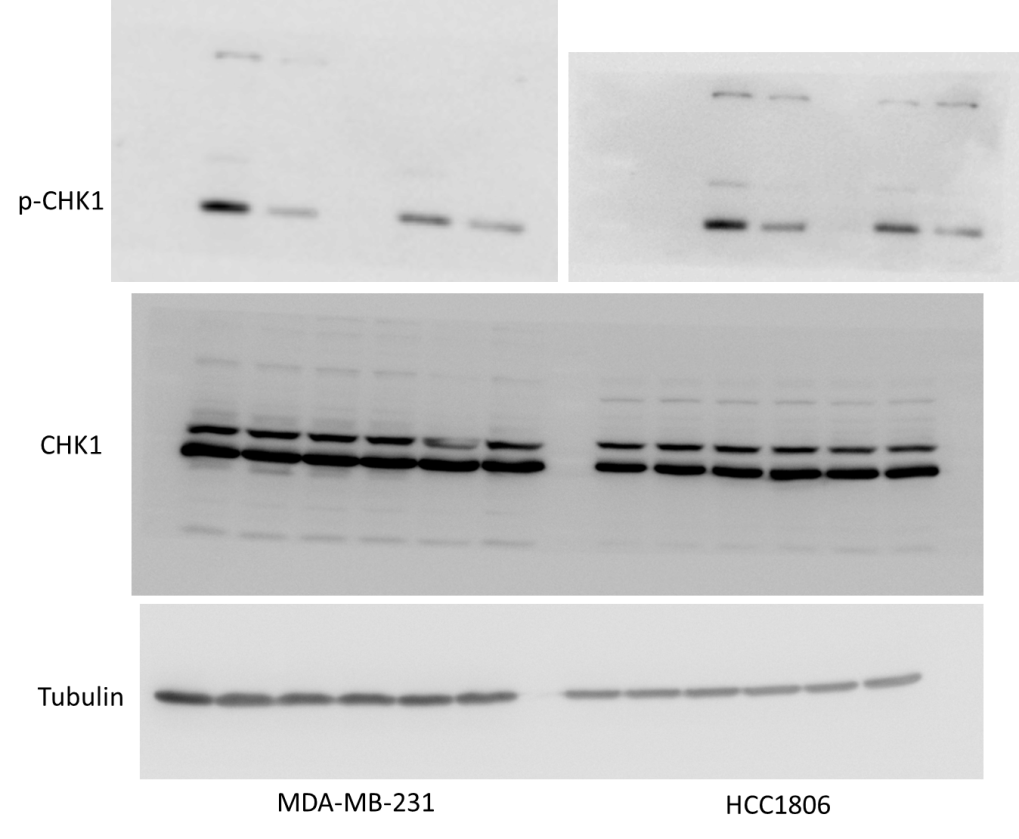


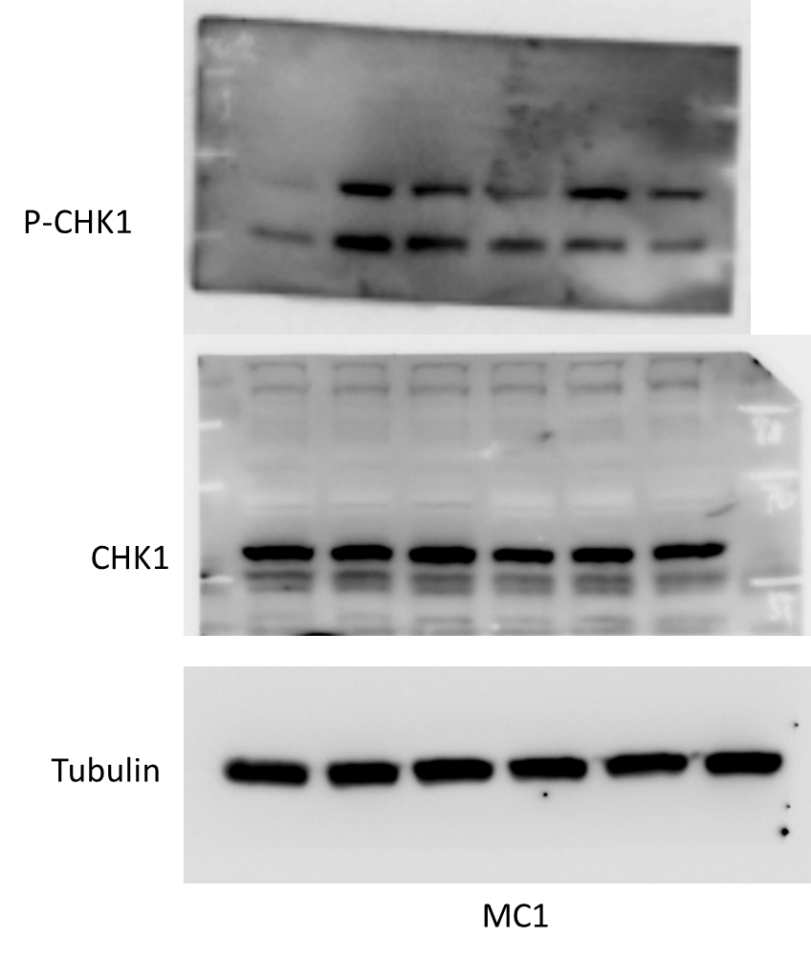


**Figure 1A**


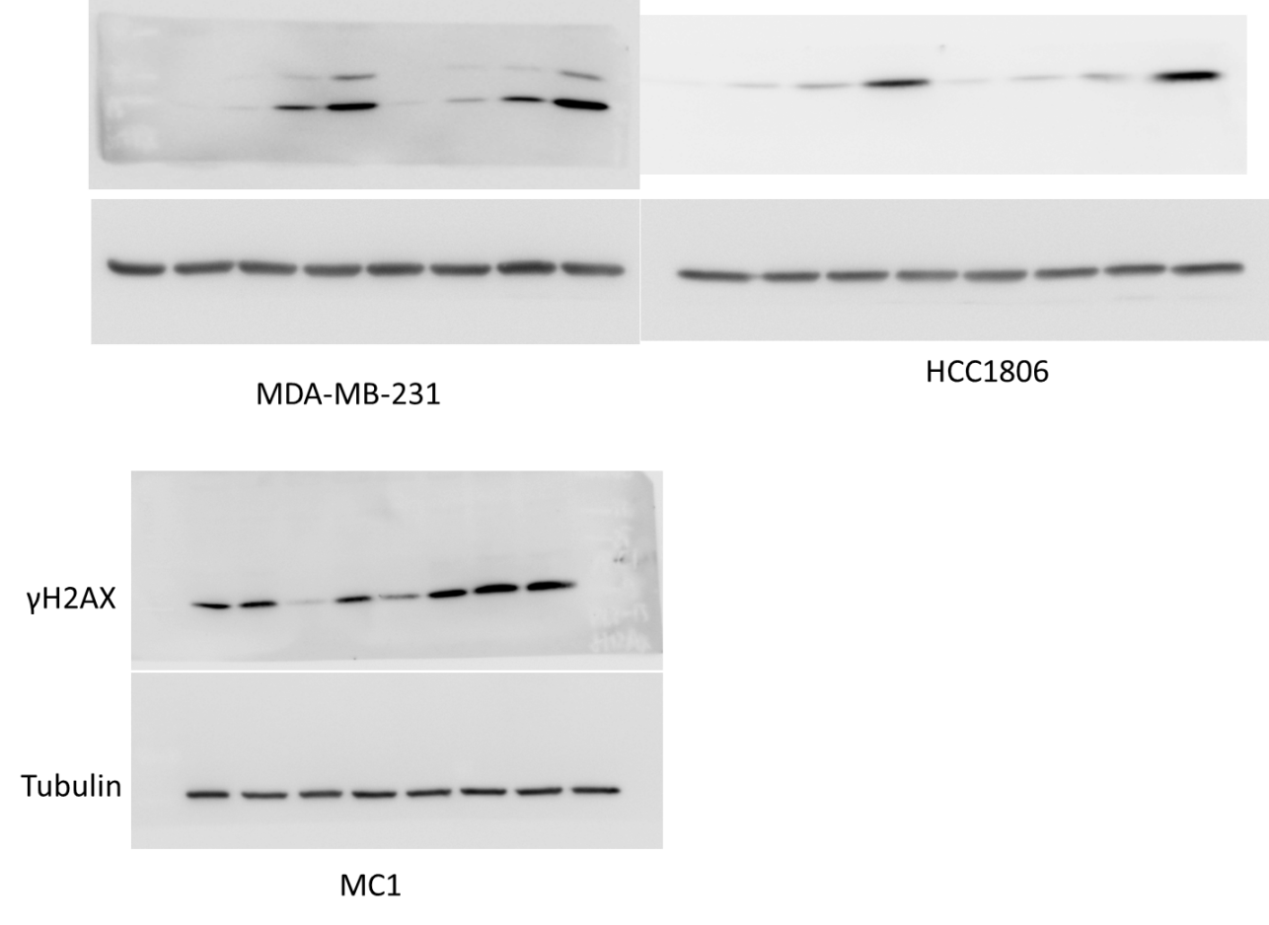


**Figure 1B**


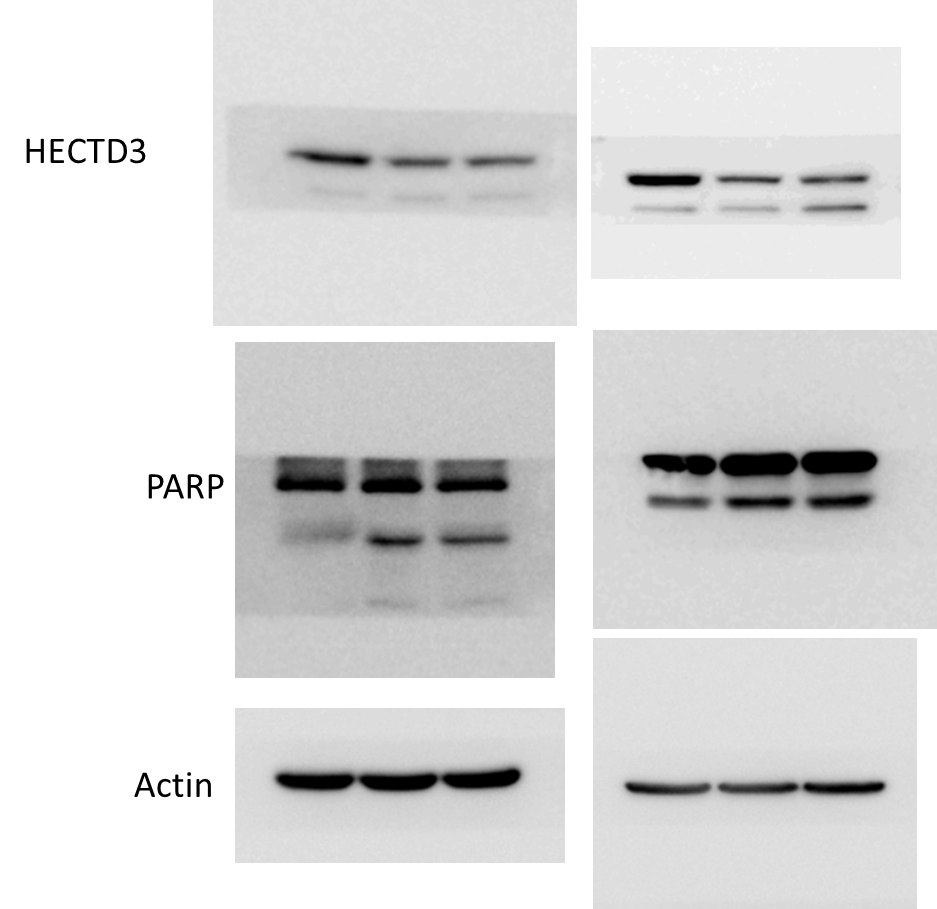


**Figure 2B**


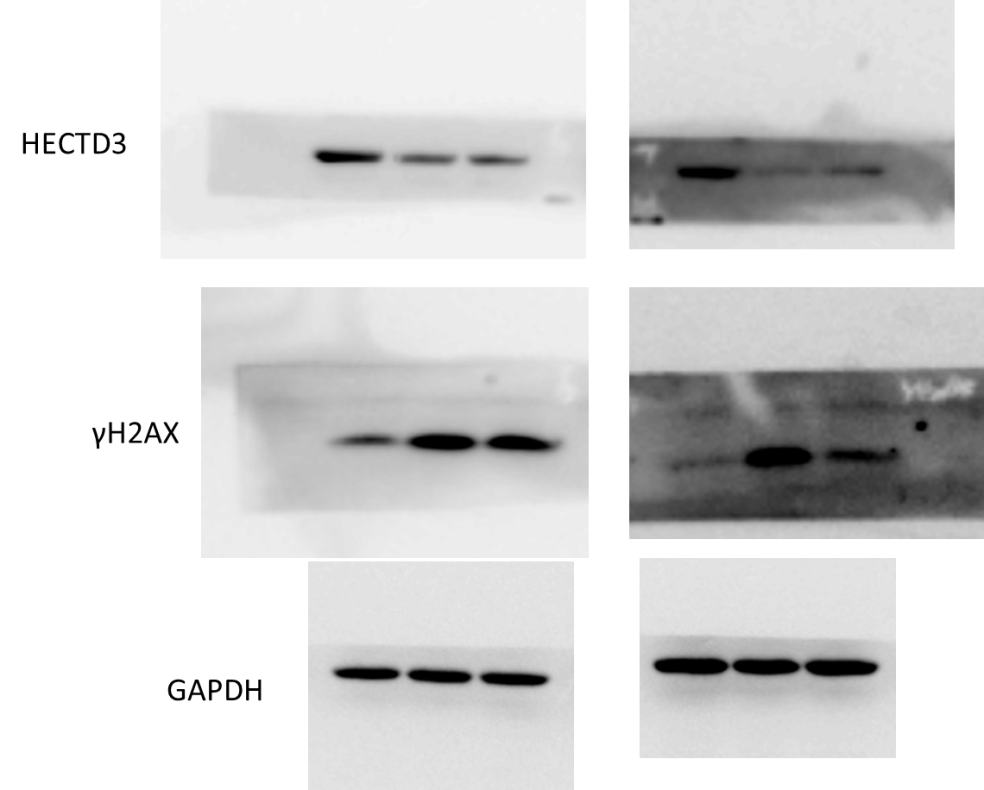


**Figure 2D**


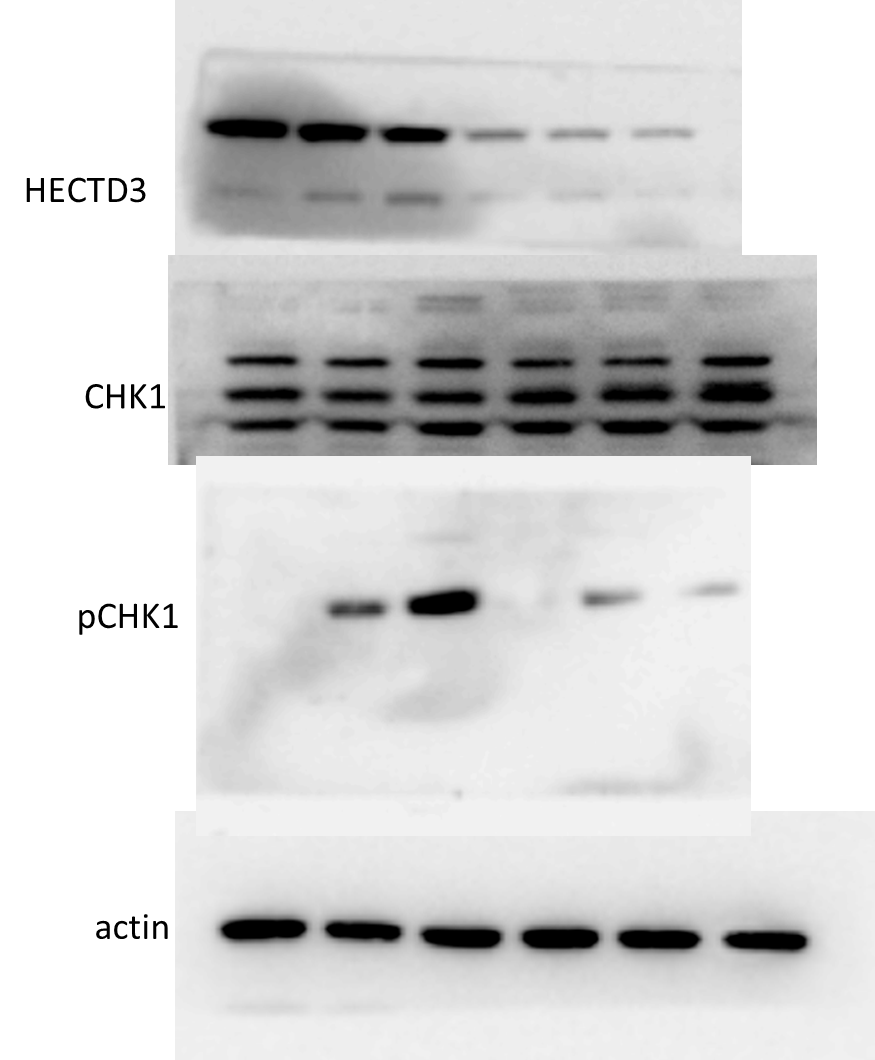


HCC 1937

**Figure 3A**


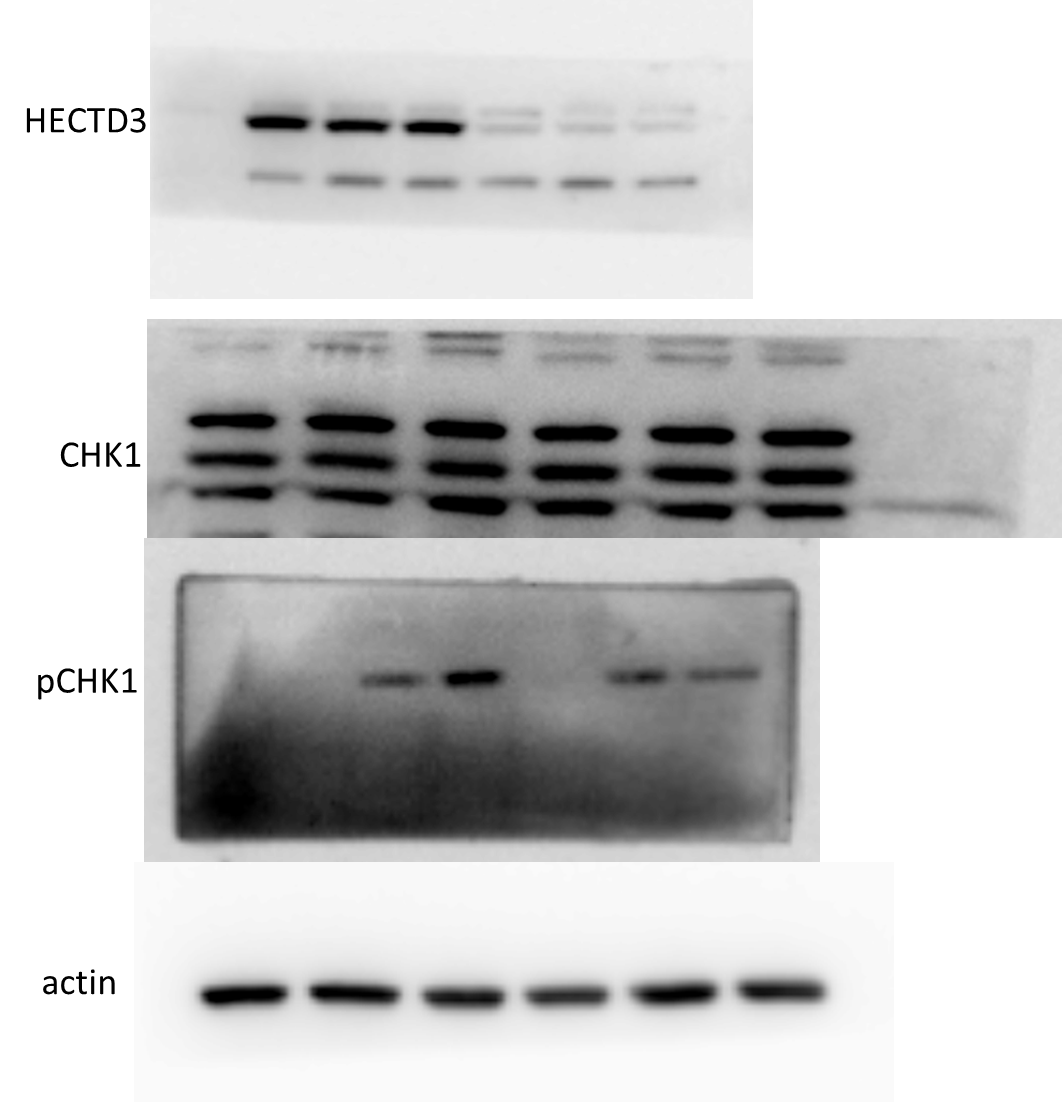


HCC1806

**Figure 3A**


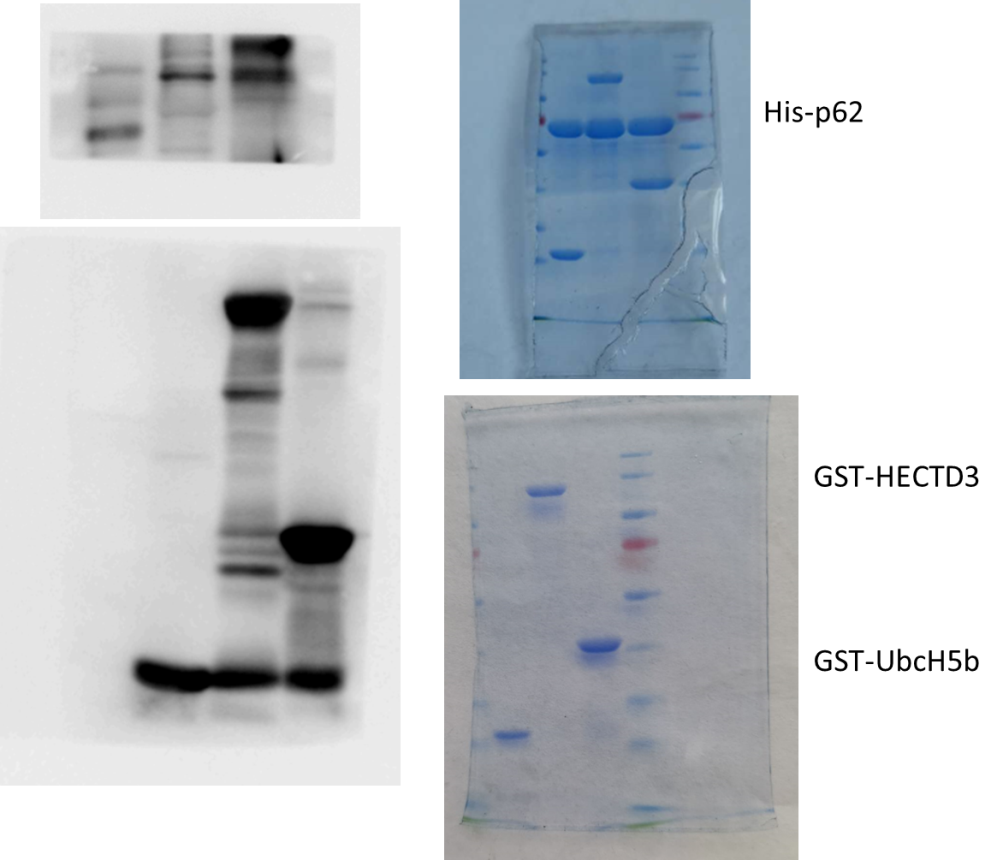


**Figure 4B**


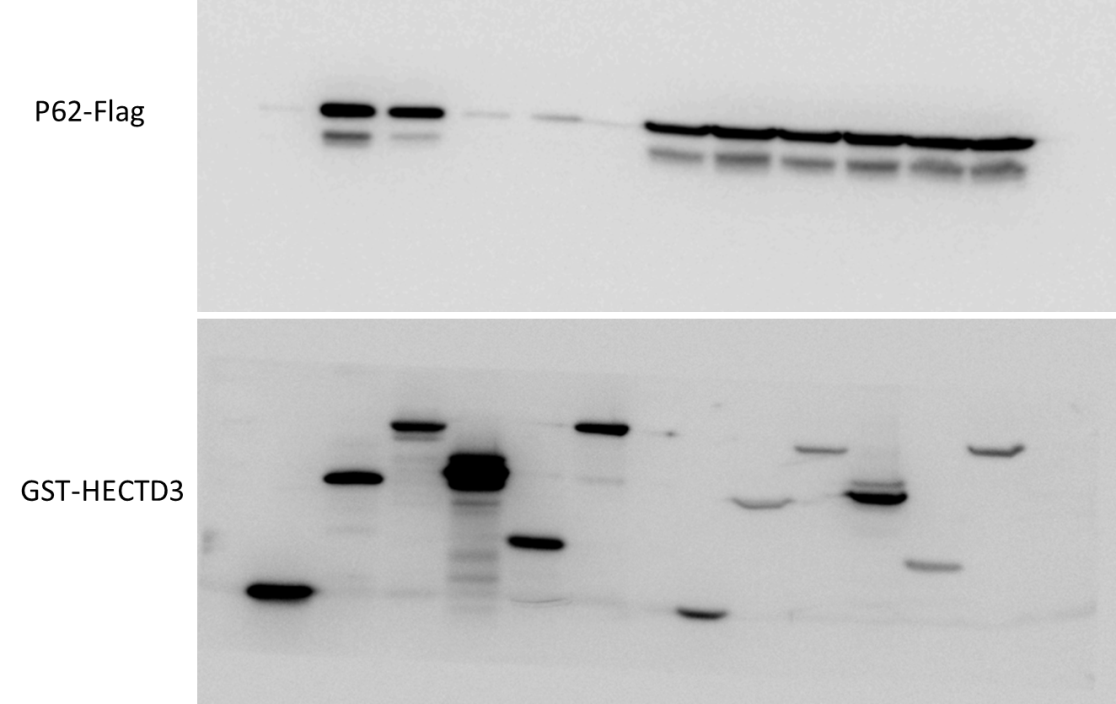


**Figure 4D**


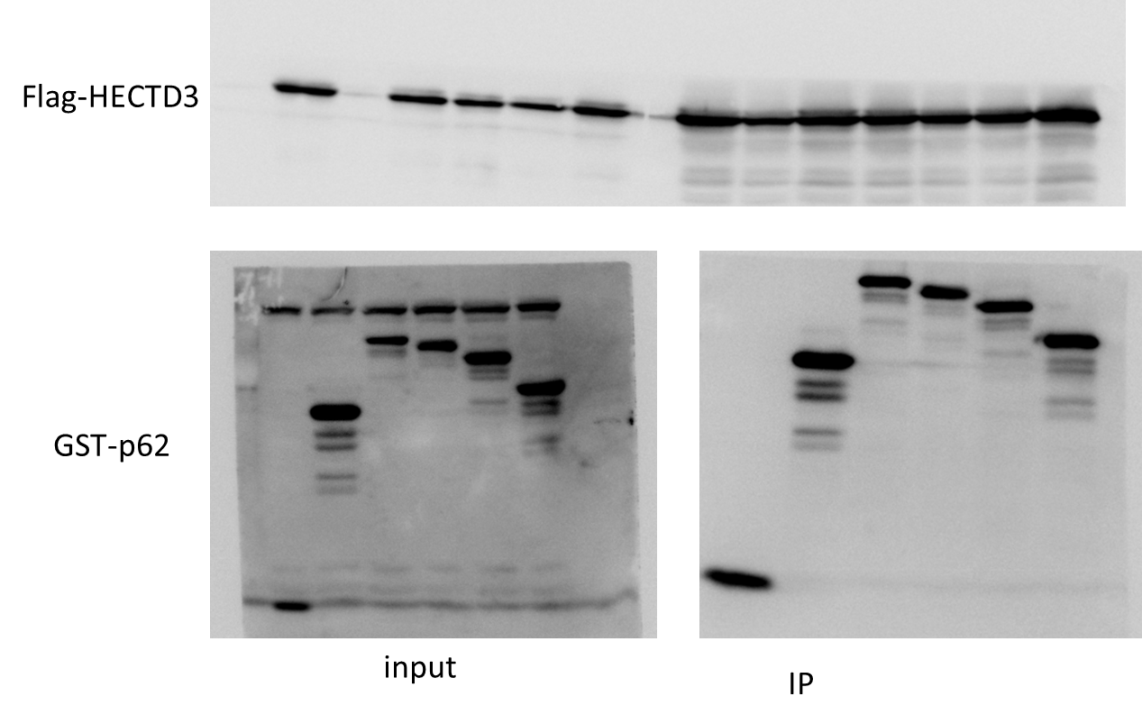


**Figure 4F**


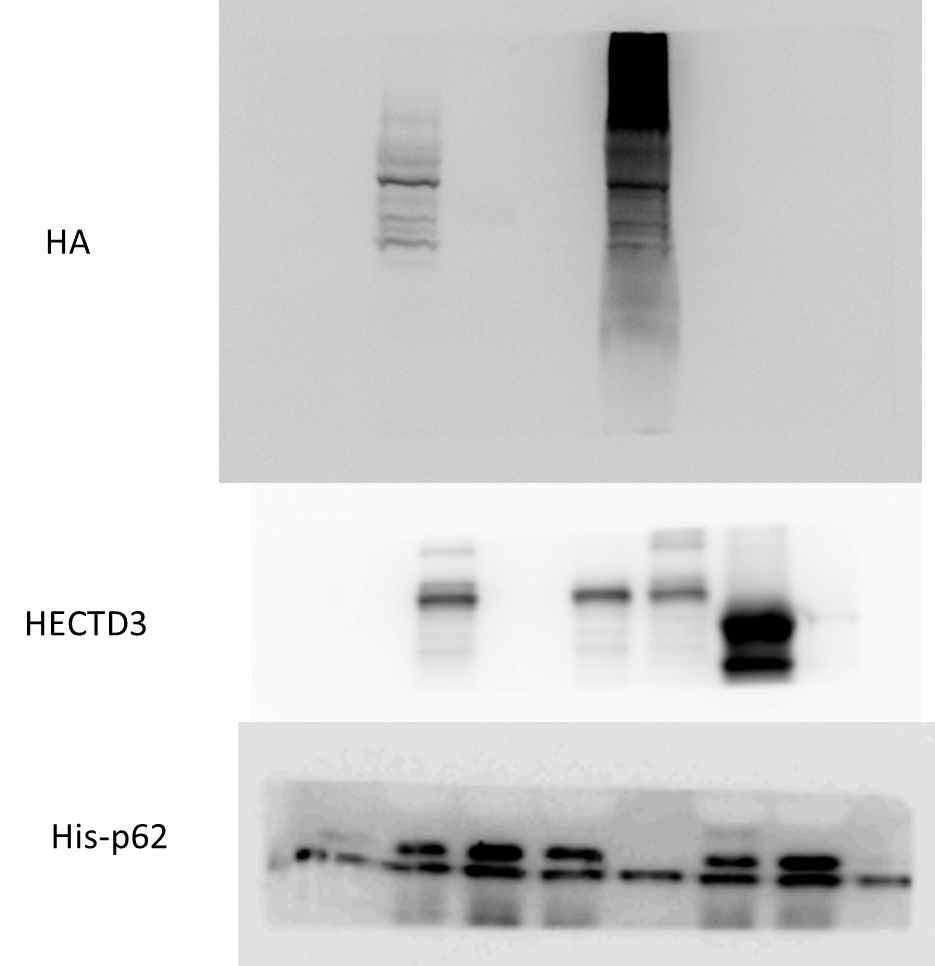


**Figure 4G**


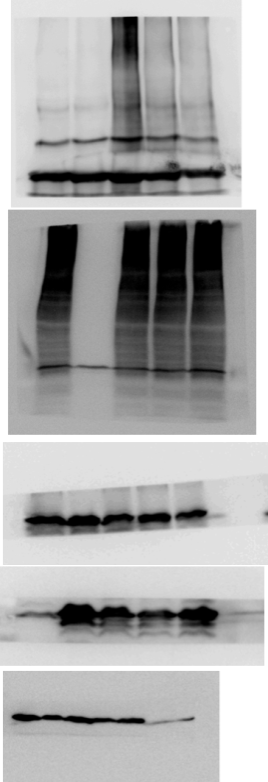


**Figure 4H**


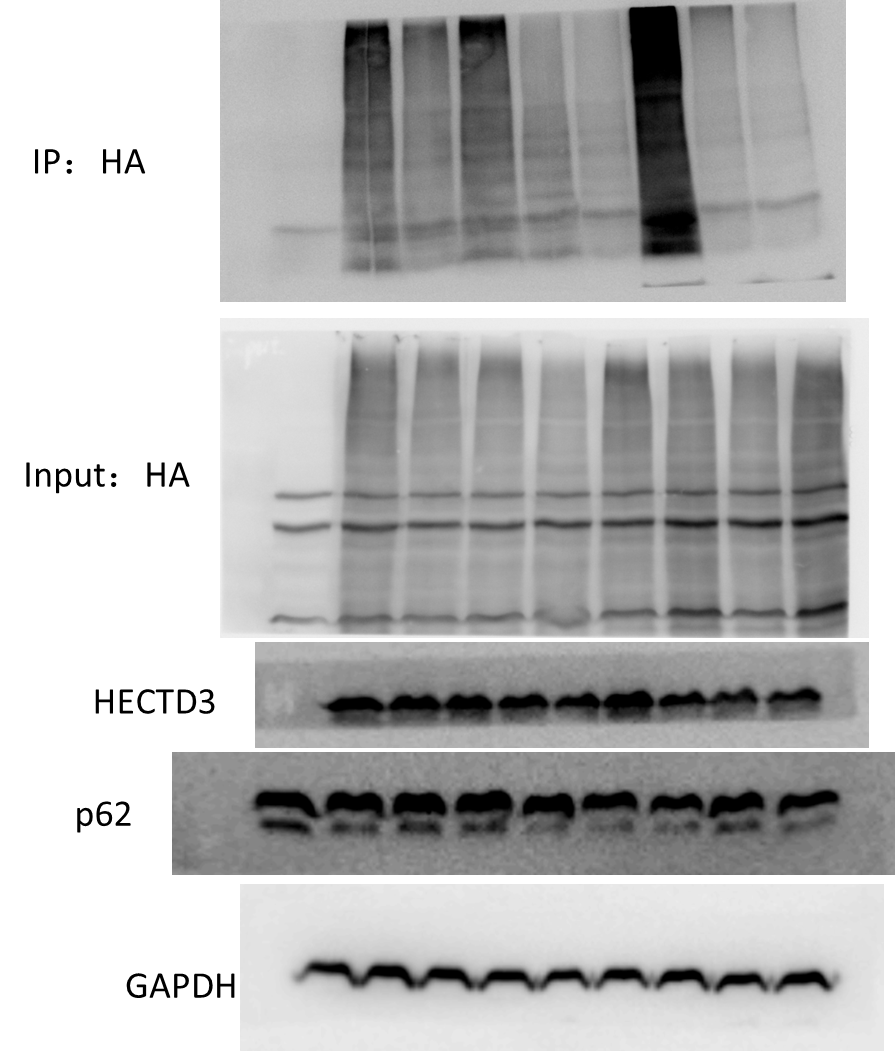


**Figure 4I**


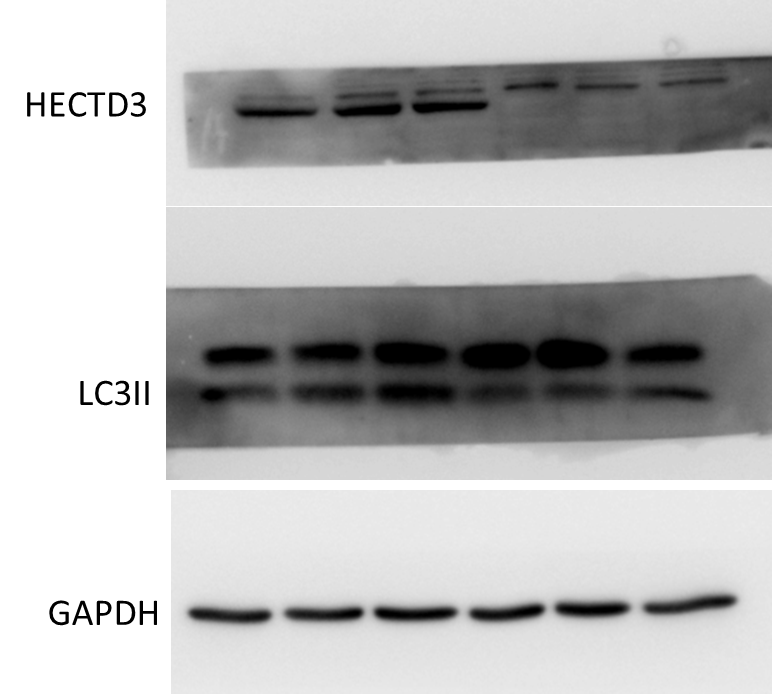


**Figure 5C**


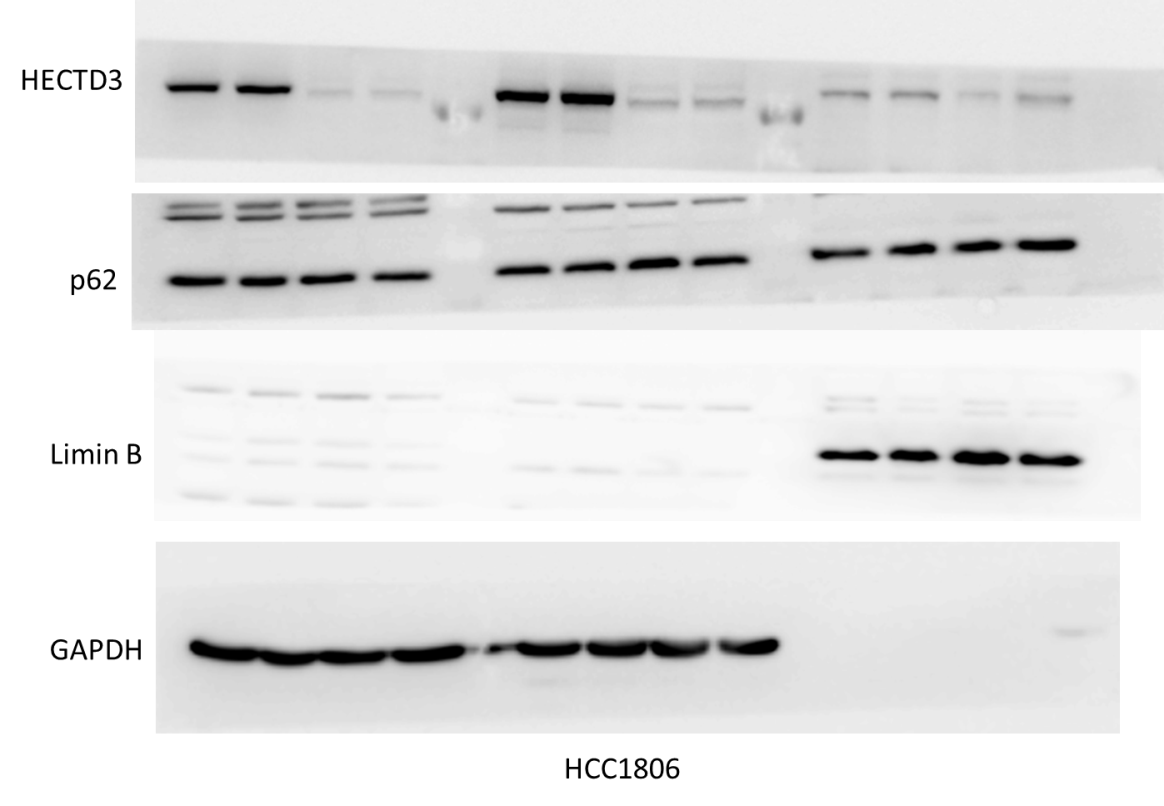


**Figure 5D**


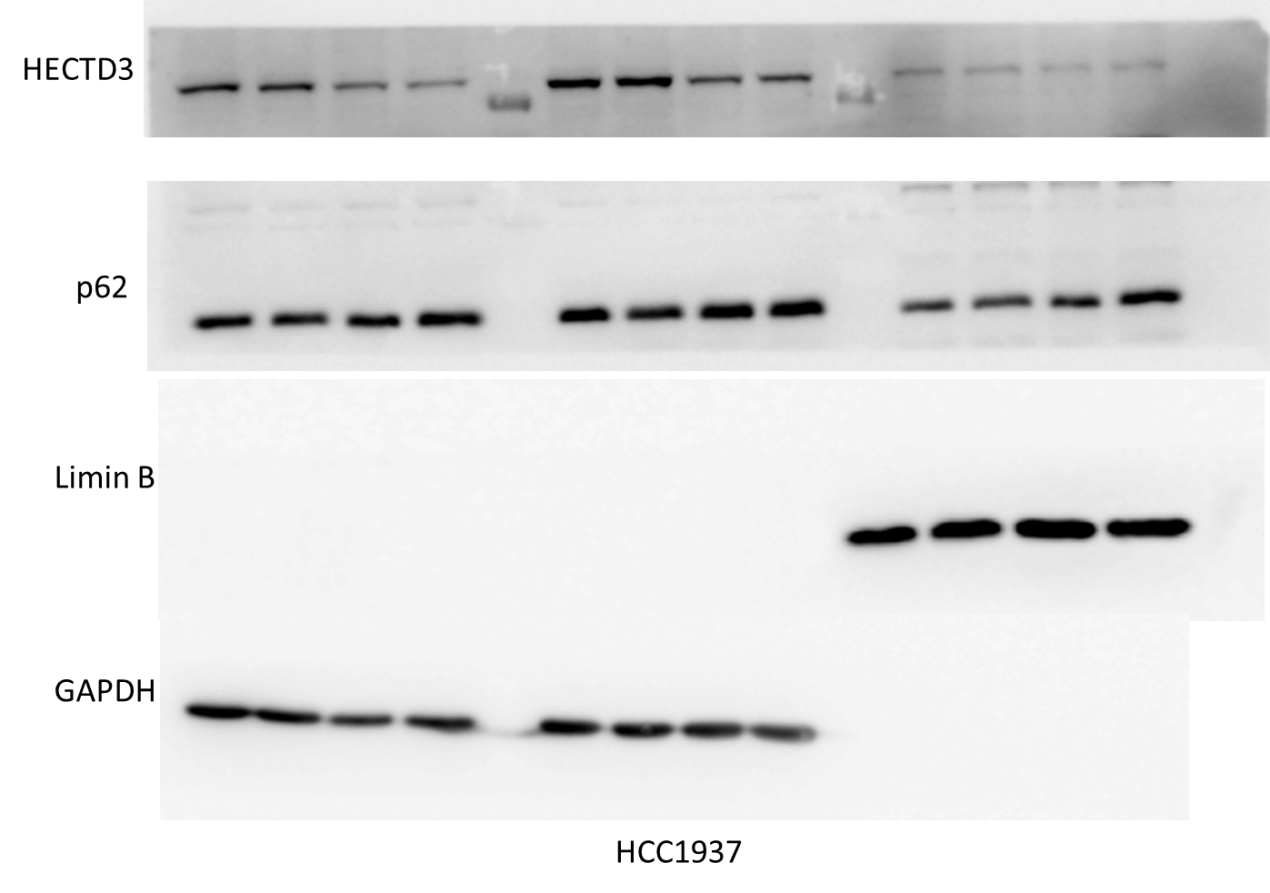


**Figure 5D**


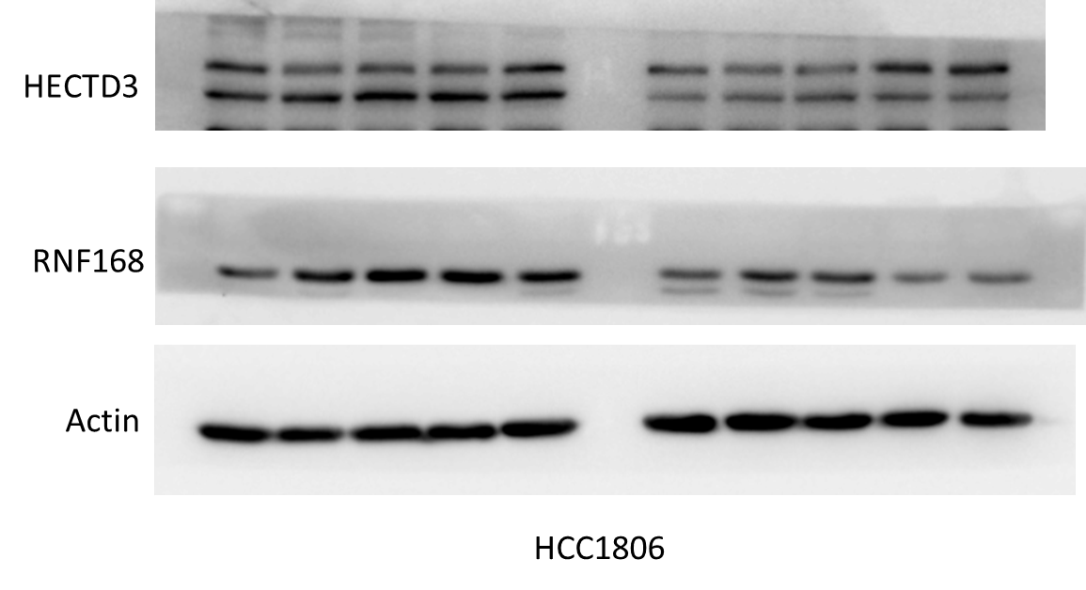


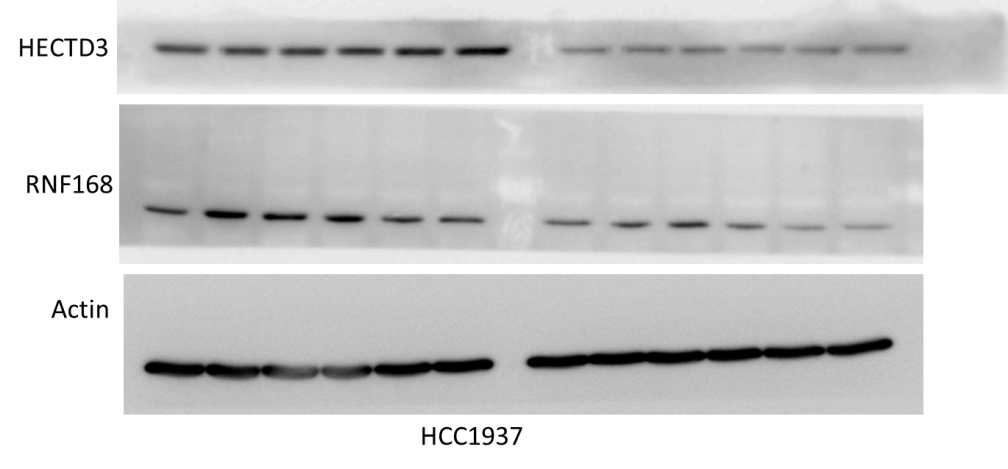


**Figure 5E**


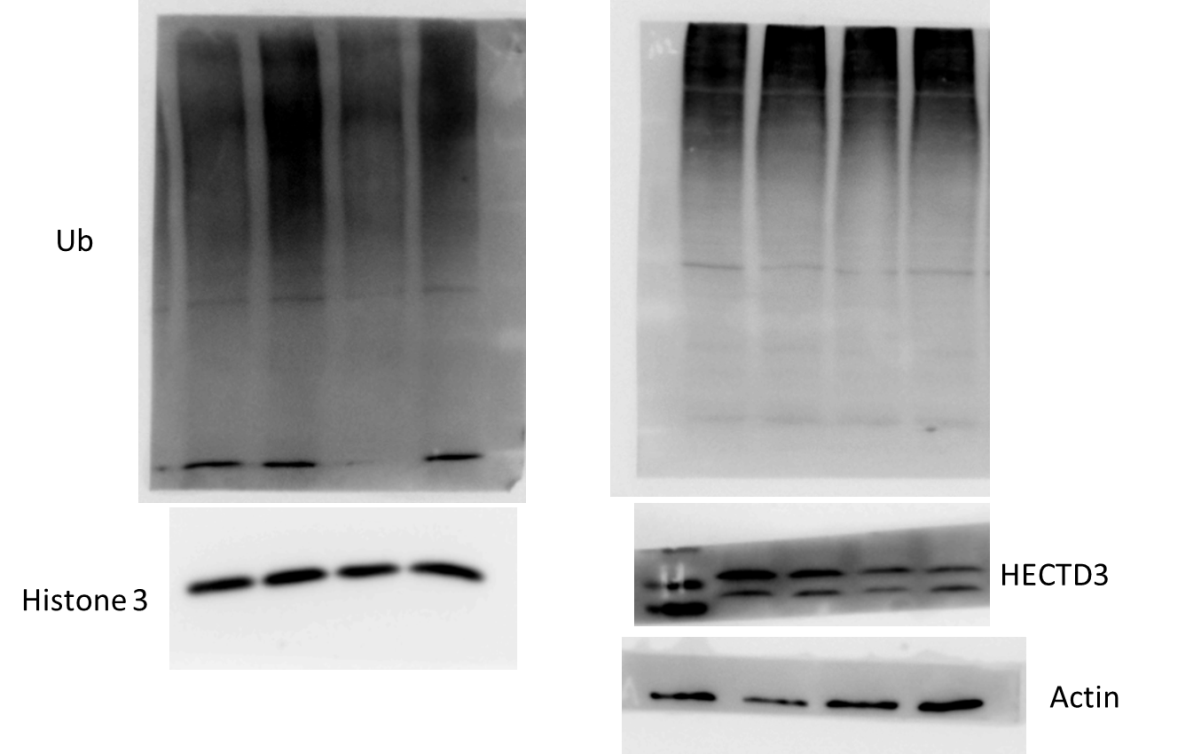


**Figure 5H**


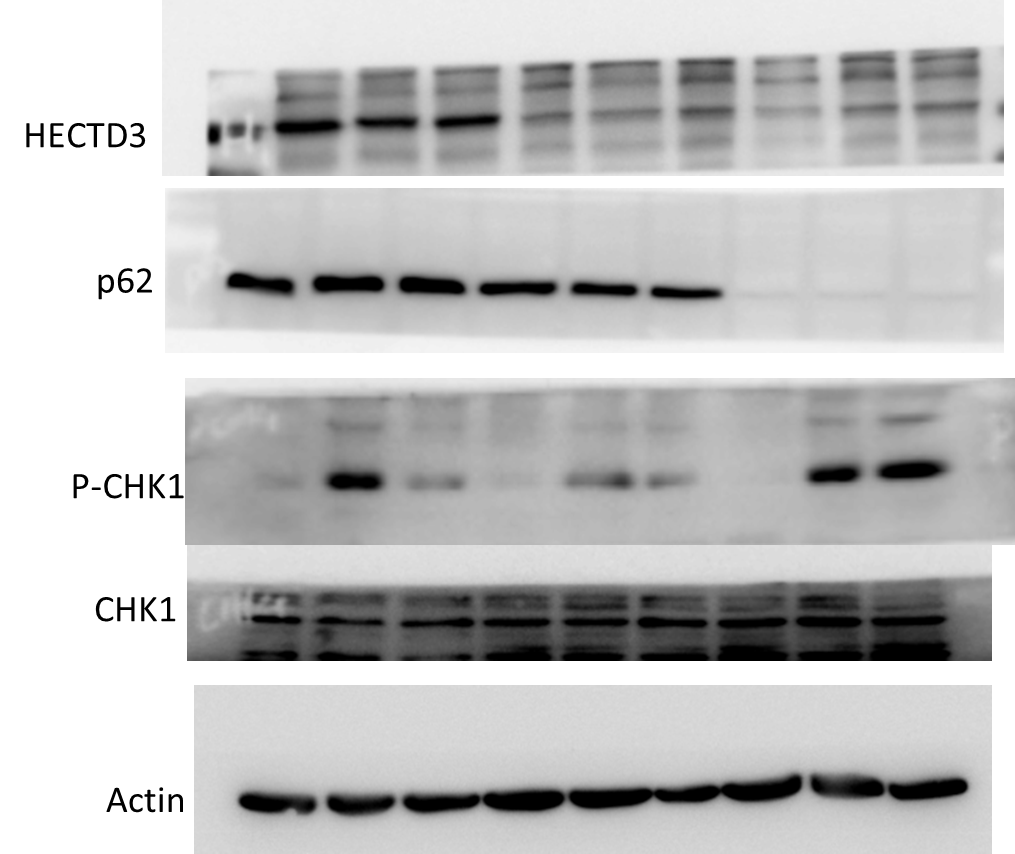


**Figure 6A**


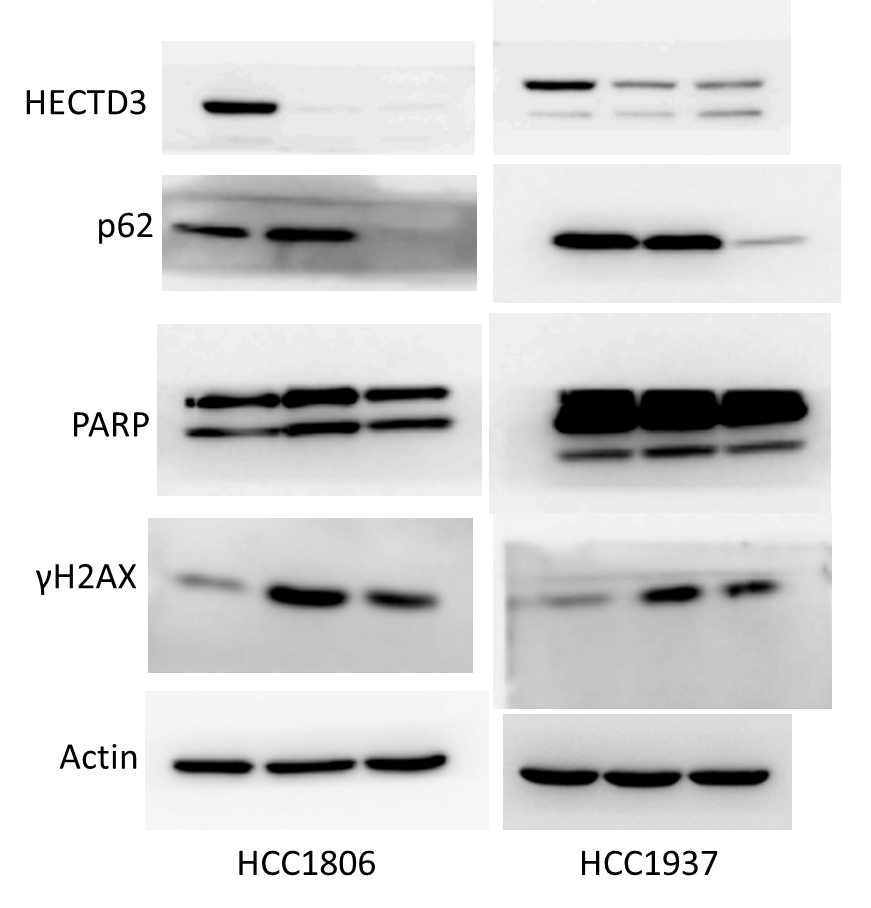


**Figure 6B**

**Reference**

[1] Li Y, Chen X, Wang Z, Zhao D, Chen H, Chen W, et al. The HECTD3 E3 ubiquitin ligase suppresses cisplatin-induced apoptosis via stabilizing MALT1. Neoplasia. 2013; 15:39-48.

[2] Li Y, Kong Y, Zhou Z, Chen H, Wang Z, Hsieh YC, et al. The HECTD3 E3 ubiquitin ligase facilitates cancer cell survival by promoting K63-linked polyubiquitination of caspase-8. Cell Death Dis. 2013; 4:e935.

[3] Zhi X, Zhao D, Wang Z, Zhou Z, Wang C, Chen W, et al. E3 ubiquitin ligase RNF126 promotes cancer cell proliferation by targeting the tumor suppressor p21 for ubiquitin-mediated degradation. Cancer Res. 2013; 73:385-94.

[4] Peng H, Yang J, Li G, You Q, Han W, Li T, et al. Ubiquitylation of p62/sequestosome1 activates its autophagy receptor function and controls selective autophagy upon ubiquitin stress. Cell Res. 2017; 27:657-74.

[5] You Z, Jiang W, Qin L, Gong Z, Wan W, Li J, et al. Requirement for p62 acetylation in the aggregation of ubiquitylated proteins under nutrient stress. Nature communications. 2019; 10:5792.

[6] Su L, Luo R, Liu Q, Su J, Yang L, Ding Y, et al. Atg5- and Atg7-dependent autophagy in dopaminergic neurons regulates cellular and behavioral responses to morphine. Autophagy. 2017; 13:1496-511.

[7] Qin J, Zhou Z, Chen W, Wang C, Zhang H, Ge G, et al. BAP1 promotes breast cancer cell proliferation and metastasis by deubiquitinating KLF5. Nat Commun. 2015; 6:8471.

[8] Kong Y, Wang Z, Huang M, Zhou Z, Li Y, Miao H, et al. CUL7 promotes cancer cell survival through promoting Caspase-8 ubiquitination. Int J Cancer. 2019; 145:1371-81.

[9] Li F, Liang H, You H, et al. Targeting HECTD3-IKKα axis inhibits inflammation-related metastasis. Signal Transduct Target Ther. 2022; 7:264.

[10] Justyna Jozefczuk, Katharina Drews, James Adjaye. Preparation of mouse embryonic fibroblast cells suitable for culturing human embryonic and induced pluripotent stem cells. J Vis Exp. 2012; 64:3854.
